# Ordered Patterns of Cell Shape and Orientational Correlation during Spontaneous Cell Migration

**DOI:** 10.1371/journal.pone.0003734

**Published:** 2008-11-17

**Authors:** Yusuke T. Maeda, Junya Inose, Miki Y. Matsuo, Suguru Iwaya, Masaki Sano

**Affiliations:** Department of Physics, Graduate School of Science, the University of Tokyo, Bunkyo-ku, Tokyo, Japan; Center for Genomic Regulation, Spain

## Abstract

**Background:**

In the absence of stimuli, most motile eukaryotic cells move by spontaneously coordinating cell deformation with cell movement in the absence of stimuli. Yet little is known about how cells change their own shape and how cells coordinate the deformation and movement. Here, we investigated the mechanism of spontaneous cell migration by using computational analyses.

**Methodology:**

We observed spontaneously migrating *Dictyostelium* cells in both a vegetative state (round cell shape and slow motion) and starved one (elongated cell shape and fast motion). We then extracted regular patterns of morphological dynamics and the pattern-dependent systematic coordination with filamentous actin (F-actin) and cell movement by statistical dynamic analyses.

**Conclusions/Significance:**

We found that *Dictyostelium* cells in both vegetative and starved states commonly organize their own shape into three ordered patterns, elongation, rotation, and oscillation, in the absence of external stimuli. Further, cells inactivated for PI3-kinase (PI3K) and/or PTEN did not show ordered patterns due to the lack of spatial control in pseudopodial formation in both the vegetative and starved states. We also found that spontaneous polarization was achieved in starved cells by asymmetric localization of PTEN and F-actin. This breaking of the symmetry of protein localization maintained the leading edge and considerably enhanced the persistence of directed migration, and overall random exploration was ensured by switching among the different ordered patterns. Our findings suggest that *Dictyostelium* cells spontaneously create the ordered patterns of cell shape mediated by PI3K/PTEN/F-actin and control the direction of cell movement by coordination with these patterns even in the absence of external stimuli.

## Introduction

Cell migration, which is a fundamental cellular response in inflammatory reactions and other physiological activities, is a highly complex process that integrates many spatial and temporal cellular events [1]–[6]. Motile bacteria and most eukaryotic cells can move in a directed (directed cell migration) or spontaneous (spontaneous cell migration) fashion depending on the presence or absence of external cues.

Directed cell migration toward a soluble ligand or chemotaxis is a general property of many motile eukaryotic cells [7]. *Dictyostelium* cells extend one or more pseudopodia at a time, with the extended pseudopodia being close to the chemoattractant source. The sensing of chemotactic cues is achieved by the activation of G-protein-coupled receptors and the cytosolic regulator of adenylyl cyclase (CRAC) [8]. To polarize and migrate up the chemotactic gradient after sensing chemical cues, cells restrict filamentous actin (F-actin) polymerization and form a leading edge. This task requires the cells to amplify a shallow external chemical gradient into a steep intracellular response. A recent study has revealed that, during chemotaxis in *Dictyostelium* cells, phosphoinositide 3-kinase (PI3K) is localized at the leading edge of the cells and new pseudopodia [9]. A counteracting phosphatase of PI3K, the phosphatase and tensin homolog, (PTEN), is simultaneously localized at the rear edge of the cell [10]. This reciprocal localization of the two proteins creates a spatial internal gradient of phosphatidyl inositol tri-phosphate (PIP_3_) across the plasma membrane; the level of PIP_3_ is higher at the leading edge than at the rear edge due to the kinase activity of PI3K.

On the other hand, motile cells are able to migrate spontaneously even in the absence of external stimuli. In the absence of external cAMP, individual *Dictyostelium* cells spontaneously form actin filaments and extend 1 or 2 pseudopodia. The new pseudopodia are retracted or attached to the substrate. After the attachment of the pseudopodia to the substrate, the cell adopts a polarized morphology for a few minutes and then retracts its rear edge and moves forward. Spontaneous cell migration allows the cells to forage and explore their surroundings by balancing random and directed migrations. It is thought that *Dictyostelium* cells extend pseudopodia in a more or less random manner [7]. Recently, *Dictyostelium* cells were observed to undergo spontaneous migration through stochastic activation of both PI3K and Ras at the sites of new pseudopod formation even in the absence of either chemoattractants or functional heterotrimeric G-proteins [11], [12]. However, the fundamental question of how a cell demonstrates spontaneous migration by randomly remodeling its shape through underlying molecular interactions of PI3K/PTEN/F-actin remains unanswered. Here, in order to reveal the mechanism of spontaneous cell migration, we employed a quantitative approach based on the statistical analysis of the cell shape of single *Dictyostelium* cells. To gain a further understanding of spontaneous cell migration, we observed two different developmental states of *Dictyostelium* cells, namely the vegetative and starved states. *Dictyostelium* cells exhibit vegetative and starved states at different developmental stages, but they are able to move spontaneously in both of states. Vegetative cells are more round in shape and move more slowly than starved cells; comparing these two cell states enables us to identify common mechanisms underlying cell migration.

We analyzed the stochastic dynamics of cell shape and their coordination with cell movement by using correlation analyses such as autocorrelation functions. Correlation analysis is a useful method for identifying repeating signal patterns that are masked by noise. We observed that despite apparently random pseudopodia formation, the remodeling of the cell shape was organized into three distinct ordered patterns: elongation, rotation, and oscillation. The ordered remodeling of cell shape was correlated with PI3K-dependent F-actin polymerization. PI3K activity was required for the formation of actin-filled pseudopodia while PTEN restricted the formation of excess pseudopodia; this supports the idea that the ordered patterns are mediated by PI3K/PTEN/F-actin. Furthermore, we observed that F-actin and PTEN spontaneously localize at the leading and rear edge of the cell, respectively. The asymmetric localization of both proteins would ensure the reinforcement of the leading edge, which would facilitate the maintenance of a biased direction of cell migration. Eventually, the cells would be able explore their surroundings by switching among the ordered patterns. Our results suggest that the ordered patterns of cell shape mediated by PI3K/PTEN/F-actin result in spontaneous cell migration even in the absence of chemotactic cues.

## Results

### Ordered remodeling of cell shape in spontaneously migrating cells

**Figure 1:**
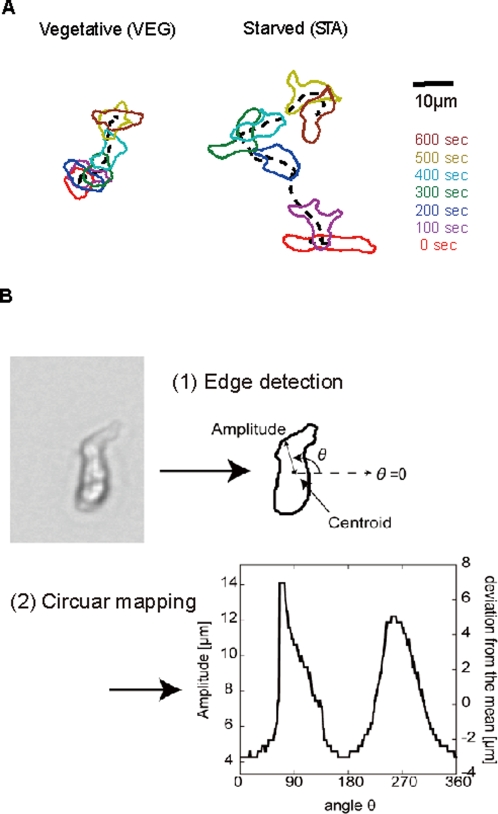
Morphological dynamics of individual cells during spontaneous cell migration. (A) Typical time-lapse images of a wild-type vegetative (WT VEG) cell and a wild-type starved (WT STA) cell. The periphery of a single cell is obtained from a binarized image. (B) Summary of Image processing. (1) edge detection and (2) circular mapping of *Amp*(*θ,t*). We define *Amp*(*θ,t*) (left ordinate) as the radial distance from the centroid to the membrane edge in the direction θ and at time t. 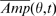 that is defined as *Amp*(*θ,t*)−〈*Amp*(*θ,t*)〉*_θ_* represents the deviation of cell shape from the average (right ordinate).

We observed single *Dictyostelium* cells in the absence of chemoattractants or nutrients at a low cell density. We made time-lapse movies over 500–600 s, at a resolution of 1 frame per second. *Dictyostelium* cells displayed two phases, a vegetative (VEG) state and a starved (STA) state (Fig. 1A). WT STA cells were highly motile and polarized with a contracted tail and a more distinct anterior lamella than WT VEG cells. These cells formed well-defined pseudopodia and crawled in an apparently random direction. The morphological dynamics of cell shape appeared to be random in both states, and initially it was difficult to discern any rules governing the changes in cell shape (Movies S1–S6).

**Figure 2:**
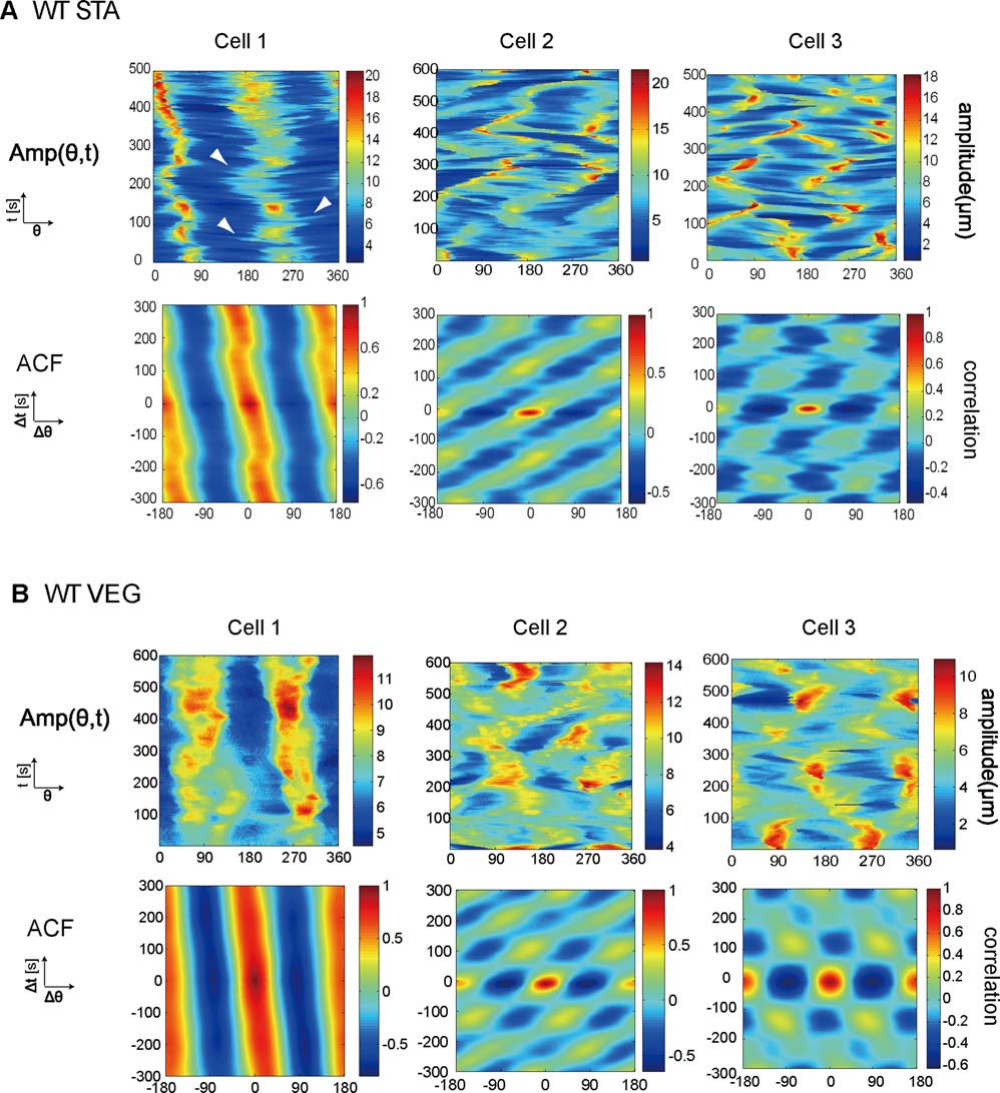
Commonly observed ordered patterns of cell shape in wild-type cells. (A) *Amp*(*θ,t*) (Upper) and the corresponding autocorrelation function (Lower) are shown for the STA cells. We show three typical examples. WT STA cell 1 (left): positive correlation persists at a certain angle with slight tilt. WT STA cell 2 (centre): propagating similar to a propagating wave. WT STA cell 3 (right): periodic with a slight tilt. White arrowhead in *Amp*(*θ,t*) of WT STA cell 1 indicates a branch generated from the leading edge. (B) *Amp*(*θ,t*) are shown for vegetative cells, with the horizontal axis θ = 0–360° and the perpendicular axis *t* = 0–500 s (WT VEG cell 2) or 0–600 s (the others) (Upper). Corresponding autocorrelation functions are also shown for WT VEG cells (Lower). WT VEG cell 1 (left): a positive correlation persists. WT VEG cell 2 (centre): propagating from one direction to another. WT VEG cell 3 (right): clearly oscillating.

First, we constructed a circular map around the centroid of a cell in order to describe the dynamic remodeling of cell shape (Fig. 1B and see Methods). We defined the variable *Amp*(*θ,t*) as the distance from the centroid to the edge of the cell membrane in the direction *θ* and at time t. *θ* = 0° was fixed in the right-hand direction on the horizontal axis. Figure 2 shows the *Amp*(*θ,t*) of both WT STA and WT VEG cells. Figure 2A shows that WT STA cells exhibit various dynamics of cell shape during spontaneous cell migration. WT STA cell 1 maintained a larger *Amp*(*θ,t*) at both 80° and 260° angles, meaning that the cell stably maintained its elongated morphology (Fig. 2A). However, it is often difficult to recognize patterns in *Amp*(*θ,t*) because of the intrinsic noise of cell deformations. In order to identify repeating patterns governing morphological dynamics, we calculated the auto-correlation function (ACF) of *Amp*(*θ,t*) (see Methods) [13]. Remarkably, we found ordered patterns (Fig. 2A, lower row). WT STA cell 1 exhibited a stable positive correlation along the lines Δ*θ* = 0° and Δ*θ* = ±180°, indicating that this cell maintains an elongated shape (elongation pattern). In WT STA cell 2, the correlation was laterally propagating, indicating the lateral propagation of pseudopodia (rotation pattern). WT STA cell 3 exhibited an oscillatory periodic profile with slight rotation; it reshaped its membrane periodically by extending pseudopodia in a certain direction and then re-extending at certain angles to the long axis of the cell in cycles of 2.5 min (oscillation pattern). Moreover, WT STA cell 1 exhibited more noisy *Amp*(*θ,t*) at the leading edge (*Amp*(260,*t*)) rather than at the rear edge (*Amp*(80,*t*)), indicating that lamellipodia are formed predominantly at the leading edge. The branches propagating from the leading edge to the rear imply that some of the lamellipodia adhere to the substrate and become fixed while the whole cell body moves forward (Fig. 2A, arrowheads). We also note that cells exhibiting a rotation or an oscillation pattern show variability of from 2.5 min to 3.5 min in the period of rotation and oscillation. We identified ordered patterns in nearly 70% of WT STA cells, and transient patterns that were intermediate between the two ordered patterns in the remaining cells (n = 53). There was no significant difference in the average area of cells exhibiting the different patterns (p>0.05, ANOVA). Our findings further suggest that WT STA cells produce three ordered patterns without the need for external stimuli.

### Ordered patterns of cell shape are common in both vegetative and starved cells

We next examined the morphological dynamics of WT VEG cells. WT VEG cells were less motile than WT STA cells, and their shapes are less polarized (Table 1). To our surprise, despite the significant decrease in migration speed and in the roundness of the cells, WT VEG cells also exhibited ordered patterns similar to those of the WT STA cells (Fig. 2B); WT VEG cells 1, 2, and 3 were deformed by elongation, rotation, and oscillation, respectively. When a cell extends pseudopodia in a certain direction and then re-extends new pseudopodia perpendicular to the long axis of the cell, an oscillation pattern occurs. 70% of the WT VEG cells exhibited the ordered patterns, and the remaining cells exhibited transient patterns that were intermediate between the two ordered patterns (n = 53). The area of a cell does not appear to determine the type of pattern since the average areas for WT VEG cells of each of the three pattern types were not significantly different (p>0.05, ANOVA). These three commonly observed patterns indicate that the morphological dynamics of WT VEG cells are also organized into ordered patterns without the need for external stimuli. The rates of occurrence of each pattern were roughly the same between vegetative and starved cells although the oscillation pattern was less frequently observed than the than other two patterns. A previous study by Killich et al. has identified rotation and oscillation patterns in WT STA cells using a different type of analysis [2]. These patterns clearly corresponded to the rotation and oscillation patterns that we observed. Moreover, our comprehensive analysis revealed that WT STA cells also show an elongation pattern and that three types of ordered pattern are commonly observed in vegetative states. Our results not only validate the previous observation but also fully characterize the dynamics of cell shape in both STA and VEG states.

**Table 1:** Parameters of spontaneous cell migration.

	Vegetative				
	WT	*pten* ^−^	WT+LY294002	*pi3k*1/2^−^	*pten* ^−^+LY294002
†Speed [µm/min]	3.96±2.01	2.50±1.03**	ND	4.01±1.05	ND
¶Roundness	0.81±0.07	0.80±0.08	0.87±0.04	0.87±0.05	0.88±0.01
‡PL [µm]	0.6	0.6	ND	0.6	ND
Elongation	16	0	0	0	0
Rotation	15	0	0	2	0
Oscillation	6	0	0	0	0
*N*	53	66	16	20	30

	Starved				
	WT	*pten* ^−^	WT+LY294002	*pi3k*1/2^−^	*pten* ^−^+LY294002
†Speed [µm/min]	15.4±5.09	3.90±1.89**	ND	4.08±1.09**	ND
¶Roundness	0.64±0.09	0.81±0.05**	0.87±0.08**	0.85±0.07**	0.89±0.02**
‡PL [µm]	16.4	0.6	ND	1.0	ND
Elongation	18	0	0	0	0
Rotation	13	0	1	2	0
Oscillation	4	0	0	0	0
*N*	53	53	22	18	33

† Cell Speed was calculated from the centre of mass displacements at 15-s time intervals. Asterisk: significantly different from WT, two-tailed Student's t-test (^**^p<0.001, ^*^p<0.05) Error indicates standard deviation. ND: not detectable

¶ Roundness that is defined as the ratio of the short and long axis of the ellipsoid is an indication of the polarity of the cells. Larger value indicates that the cells are more round and less polarized. Cell shape was approximated into the ellipsoid with the same centroid. Asterisk: significantly different from WT, two-tailed Student's t-test, (p<0.01). Error indicates standard deviation.

‡ Persistence length (PL) is the length that a cell moves in a given direction straightly. We show the number of cells exhibiting elongation, rotation, and oscillation patterns. *N* is the total number of cells we sampled. Elongation, rotation, and oscillation: the number of cells for each pattern.

**Figure 3:**
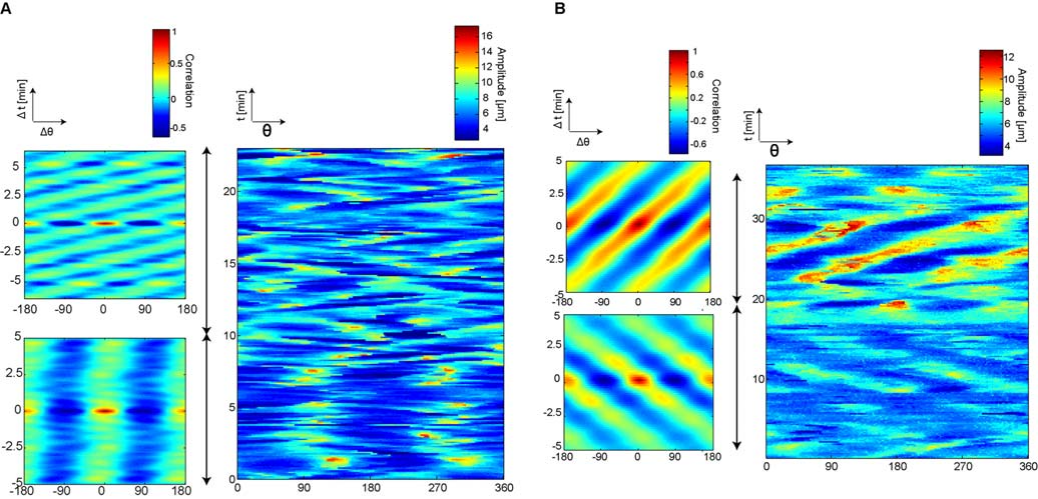
Transition of ordered patterns at a single cell. Long-term measurement for more than 20 min reveals that individual cells switch ordered patterns spontaneously. We showed *Amp*(*θ,t*) and corresponding ACFs to the left of *Amp*(*θ,t*). Black arrow indicates the calculation range of each ACF. (A) WT STA; elongation (0 to 10 min) and rotation (10 to 20 min) (B) WT VEG; the right-handed rotation pattern (2 to 20 min) and the left-handed rotation pattern (20 to 30 min).

The fact that certain patterns were commonly observed in the different cell types raise a question: whether the transition of ordered patterns occurs or not in a single cell. To answer this question, we performed long-term observation of single WT cells for more than 30 min. We then calculated the ACF of long-term *Amp*(*θ,t*) data by averaging over moving windows of 10 min. Figure 3A shows that the WT STA cell exhibited the elongation pattern until 10 min, then switched to the 72 degree/min rotation pattern. Figure 3B shows that the WT VEG cell also exhibited the transition between ordered patterns, but in this case as a change from a right-handed rotation pattern of 36 degree/min (2 to 20 min) to a left-handed rotation pattern of 36 degree/min (20 to 30 min). Moreover, 3.3 hour measurement of a WT vegetative cell revealed that a single cell can show three types of pattern, a rotation, elongation, and oscillation pattern (Fig. S1). However, when we calculated the ACF of this cell by averaging the *Amp*(*θ,t*) data over 3.3 hour, we no longer observed ordered patterns (data not shown). These results indicate that the cells showed ordered patterns lasting for 10 to 20 min and that the patterns then changed spontaneously via the stochastic transition of the patterns.

### Ordered patterns of cell shape are mediated by PI3K and PTEN

We demonstrated that morphological dynamics are spontaneously organized into three ordered patterns. What kind of molecule(s) controls these patterns? In order to address this question, we explored the specific molecular events that affect the patterns. We focused on two molecules, PTEN and PI3K. PTEN is a lipid phosphatase that dephosphorylates phosphatidylinositol-3,4,5-trphosphate (PIP_3_) to phosphatidylinositol-4,5-biphosphate (PIP_2_), whereas PI3K is a counteracting kinase that reciprocally converts PIP_2_ to PIP_3_. Reciprocal regulation of PIP_3_ by PI3K and PTEN involves in maintaining cell polarity and in efficient chemotaxis [7], [14]–[20]. The PI3K pathway is also linked to the control of F-actin polymerization mediated by RacB activation and the RacGEF1 translocation to the plasma membrane [21]–[23]. The PI3K-dependent F-actin polymerization is essential for reinforcing the leading edge during chemotaxis. However, apart from their association with cell polarity and chemotaxis, how the PI3K pathway acts cooperatively to control both the dynamics of cell shape and its coordination with spontaneous migration is poorly understood.

**Figure 4:**
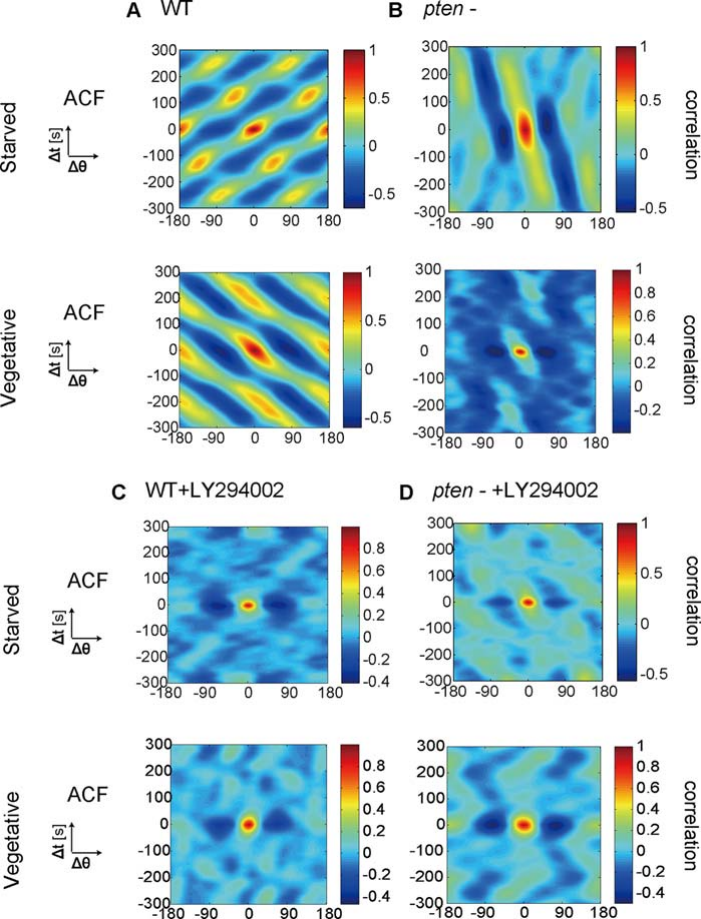
Ordered patterns of morphological dynamics are disorganized following knockdown of PTEN and following inhibition of PI3K. Typical ACFs of *Amp*(*θ,t*) are shown for (A) WT, (B) *pten* ^−^, (C) WT+LY294002 and (D) *pten* ^−^+LY294002. Upper: the STA state, Lower: the VEG state. LY294002 is a specific inhibitor of PI3K.

We initially examined the morphological dynamics of the *pten* null mutant (*pten* ^−^). *pten* ^−^ cells migrated more slowly than WT cells in both the VEG and STA states, and exhibited a less polarized shape with broad pseudopodia (Table 1, Fig. S2, Movies S7 and S8). We found much less ordered patterns in *pten* ^−^ cells in either the VEG or STA states compared with WT cells (n = 66 in VEG and n = 53 in STA; Figs 4A and 4B). *pten* ^−^ cells were defective in the spatially restricted formation of pseudopodia and failed to organize the patterns, probably because the absence of 3-phosphatase activity by PTEN leads to an increase in the proportion of the membrane area containing proteins with a PH domain, and rapid and erratic expansion of the region from which actin-filled pseudopodia are extended [7], [10], [16], [24]. Thus, PTEN is a key regulator for organizing morphological dynamics by suppressing excess pseudopodia in both the VEG and STA states. We therefore investigated the morphological dynamics of WT cells treated with the specific inhibitor LY294002 (PI3K-inhibited cells) (n = 16 in VEG and n = 22 in STA) and the *pi3k1/2* null mutant (n = 20 in VEG and n = 18 in STA). The PI3K-inhibited cells exhibited an aberrant cell shape and were poorly polarized (Movies S9–S12), and rarely displayed ordered patterns in both the VEG and STA states (Fig. 4C); 5% of PI3K-inhibited cells exhibited a rotating pattern, which may have been due to either the incomplete inhibition of PI3K activity or to redundant *pi3k* genes. In order to further clarify the effect of PI3K and PTEN further, we examined the morphological dynamics of the double mutant, *pten* ^−^+LY294002 cells. This mutant also exhibited a less polarized cell shape with a single (or sometimes multiple) pseudopodium and less motility (Movies S13 and S14). We did not observe ordered patterns in the double mutant, suggesting that the timing and the direction of pseudopodia became to be random due to the inhibition of both PI3K and PTEN (n = 30 in VEG and n = 33 in STA; Fig. 4D). Hence, PI3K and PTEN but not external signals are required to induce ordered remodeling of the cell shape from random morphological dynamics in both the developmental stages.

### PI3K-dependent actin polymerization correlates with ordered patterns

**Figure 5:**
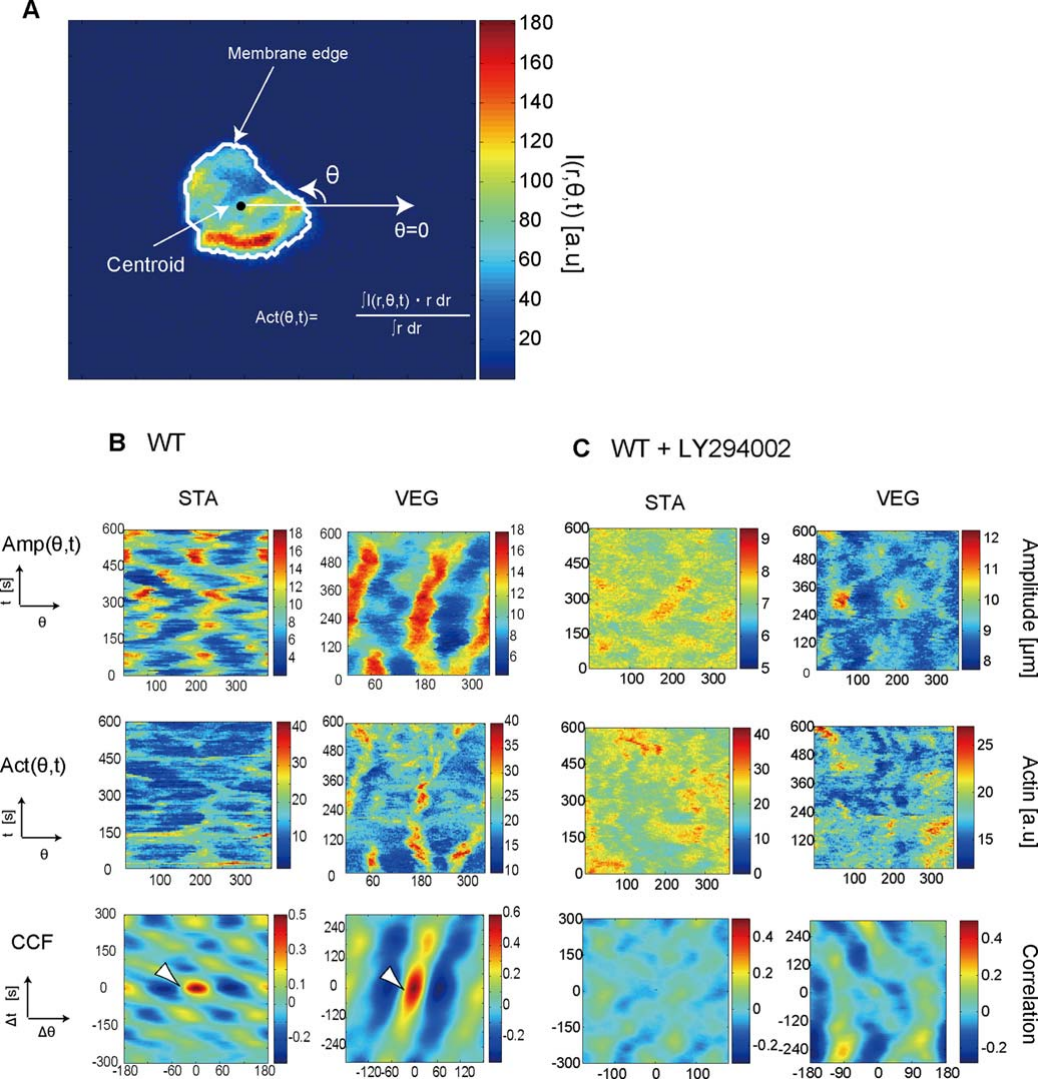
F-actin accumulation correlates with membrane elongation. (A) Summary of the analysis of F-actin accumulation. Scale bar is 20 µm. (B) and (C): *Amp*(*θ,t*), *Act*(*θ,t*) and the cross-correlation function (CCF) *C_Act,Amp_*(Δ*θ*,Δ*t*) between *Amp*(*θ,t*) and *Act*(*θ,t*) for the WT cells in both VEG and STA (B) and for the WT+LY294002 cells in both VEG and STA (C). White arrowhead in CCF of WT cells indicates the high degree of correlation between the dynamics of F-actin accumulation and the dynamics of cell shape.

We next considered the specialized role of PI3K and PTEN in the control of cell shape. In order to gain insight into the PI3K-dependent organization of morphological dynamics at the molecular level, we investigated the dynamics of PI3K-dependent F-actin polymerization in both WT VEG and WT STA cells. Measurement of actin binding domain fused GFP (ABD-GFP) expression was used as an index of F-actin assembly and localization [25] (Fig. 5A). We measured the dynamics of F-actin accumulation (*Act*(*θ,t*)) and the dynamics of cell shape (*Amp*(*θ,t*)) every 6 s, and examined the correlation between these processes using a cross-correlation function (CCF; see Methods). We found that WT cells showed a large correlation between *Act*(*θ,t*) and *Amp*(*θ,t*) in both the VEG and STA states, suggesting that F-actin was predominantly accumulated in the elongated region of the cell (Fig. 5B). In contrast, PI3K-inhibited cells exhibited fast-decaying accumulation of F-actin but no significant correlation between *Act*(*θ,t*) and *Amp*(*θ,t*) in both the VEG and STA states (Fig. 5C). The high degree of cross-correlation suggests that PI3K activity regulates the amplitude of pseudopodia through PI3K-dependent actin polymerization.

In addition to the CCF between *Amp*(*θ,t*) and *Act*(*θ,t*), we further examined the specialized role of PI3K and PTEN in the control of cell shape by using power spectrum of *Amp*(*θ,t*). The power spectrum of *Amp*(*θ,t*) provides additional information than the CCF on the mechanism underlying the development of ordered patterns because it is a measure of the amplitude of pseudopodia at scales of different length. We found that the spectrum of PI3K-inhibited cells had lower amplitude, suggesting that PI3K-inhibited cells rarely grow large pseudopodia (Fig. S3). Moreover, a small spectrum amplitude is a commonly observed property in both the VEG and STA states. These results are consistent with the strong correlation of the CCF in WT cells (Fig. 5). In addition, the amplitude of the spectrum of *pten* ^−^ cells was comparable to that of the WT cells, suggesting that PTEN disruption did not affect the amplitude of pseudopodia (Figs. S2 and S3). It appears that PTEN does not control the amplitude of pseudopodia but is essential for the formation of spatially restricted pseudopodia as a suppressor of random pseudopodial activity. Furthermore, treatment of *pten* ^−^ cells with LY294002 also resulted in a spectrum of smaller amplitude. These results support our conclusion in the preceding section: the initiation of an ordered remodeling of cell shape from random morphological dynamics is mediated by both the PI3K-dependent actin polymerization and the suppression of excess pseudopodia by PTEN.

### Coordination between ordered remodeling of cell shape and cell movement

**Figure 6:**
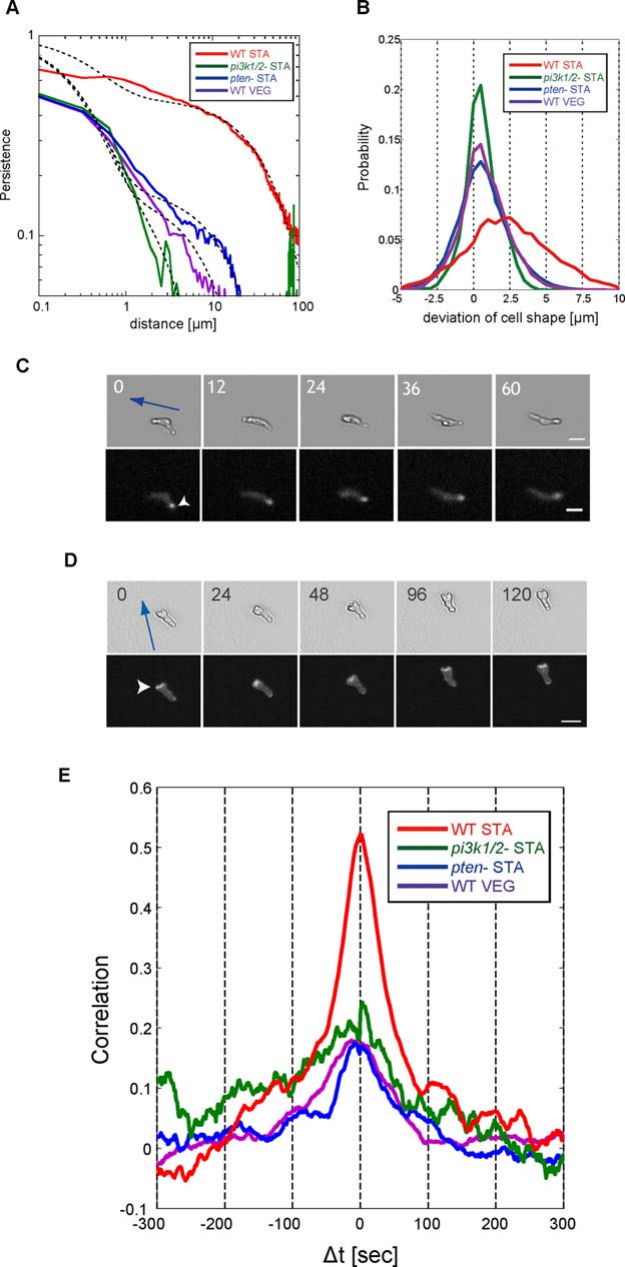
Coordination of cell movement with ordered patterns. (A) Persistence of directional motion as a function of distance in WT cells and mutants. Slow decay of persistence represents the sustainable straight motion. Red: WT STA cells, Purple: WT VEG cells, Blue: *pten* ^−^ STA cells, Green: *pi3k*1/2^−^ STA cells. The curve of persistence is well fitted by the double exponential function (black dash line). (B) Spontaneous symmetry breaking of cell polarity. Probability distribution of the deviation of cell shape of the direction of migration (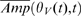). Red: WT STA cells, Purple: WT VEG cells, Blue: *pten* ^−^ STA cells, Green: *pi3k*1/2^−^ STA cells. (C) and (D): Spontaneous asymmetric localization of (C) PTEN and (D) F-actin. Blue arrow denotes the direction of cell movement. White arrowhead denotes the localization area. Scale bar is 20 µm. The number indicates time of measurement [second]. (E): Identical angle cross-correlation function between *Amp*(*θ,t*) and *V*(*t*). Red: WT STA cells, Purple: WT VEG cells, Blue: *pten* ^−^ STA cells, Green: *pi3k*1/2^−^ STA cells.

Finally, we addressed a central problem of cell migration, the relationship between morphological dynamics and cell migration. We defined the persistence length of centroid motion as a characteristic length of persistence of directional migration (see Methods).. The persistence of directional migration of WT STA cells gradually decays as a function of distance, while that of both *pten* ^−^ STA and *pi3k*1/2^−^ STA cells, and also of WT VEG cells, rapidly reaches to 0 (Figs. 6A and S4, Text S1); The averaged persistence length of WT STA cells (16.4 µm) was 27 times greater than those of both *pten* ^−^ STA and *pi3k*1/2^−^ STA cells, and also that of WT VEG cells (Table 1). The difference of persistence length is not explained solely by the difference of velocity because WT STA cells move four times faster than WT VEG cells. In order to reveal the mechanism by which WT STA cells create persistent motion in the absence of external signals, we investigated the coordination between cell shape and cell movement. We calculated the probability distribution of the deviation of *Amp*(*θ,t*) in the direction of migration, 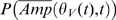 (see Methods). This distribution tells us whether a cell tends to extend (or retract) its membrane in the direction of migration. The probability distribution of WT STA cells was largely biased towards the positive side (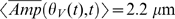), whereas that of both *pten* ^−^ STA and *pi3k*1/2^−^ STA cells, and also of WT VEG cells, was only slightly shifted toward the positive side with 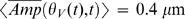 (Fig. 6B). The bias of WT STA cells was approximately 6 times larger than that of the other cell. The difference in distribution profiles confirms that WT STA cells directly migrate in the direction of protrusions without the necessity of external cues, while PTEN disruption or PI3K1/2 deletions diminished such coordination between cell polarity and cell migration. Furthermore, we observed the spatial localization of PTEN-GFP and ABD-GFP and found that PTEN and F-actin were spontaneously localized at the rear and the leading edges asymmetrically in WT STA cells, respectively [12] (Figs. 6C and 6D). We did not observe the asymmetric localization of PTEN-GFP at the membrane in WT VEG cells. In addition, the localizations of these molecules were abolished by inhibition of actin polymerization either by latrunculinA or by LY294002 (data not shown), indicating that actin polymerization is required for establishing asymmetric localization of PTEN. Our observations imply that the breaking of spontaneous symmetry accompanied by localization of PTEN reinforces the cell polarity and that the marked change in persistence length between the VEG and STA states is controlled through the persistent asymmetric localization of F-actin mediated by the PI3K pathway. Furthermore, we evaluated the correlation between the membrane extensions (or retractions) along the direction of cell movement, *Amp*(*θ,t*) at time *t = t+Δt* and the centroid displacements *V*(*t*) at time *t = t* by using the identical angle cross-correlation function (iaCF) as follows (see Methods); the iaCF is useful for evaluating the coordination between cell deformation and cell movement for individual cells. When we obtain the maximum value of the iaCF at *Δt = τ*, this cell tends to protrude its own membrane toward the direction of cell movement with a time delay of *τ*. We found four times larger positive correlation in the WT STA compared with the WT VEG cells, *pten* ^−^ STA cells, and *pi3k*1/2^−^ STA cells at *Δt = 0* (Fig. 6E). This result indicates that the WT STA cells coordinate the extension and retraction of protrusions with cell movement and that the PI3K inhibition and *pten* depletion causes the loss of such coordination.

**Figure 7:**
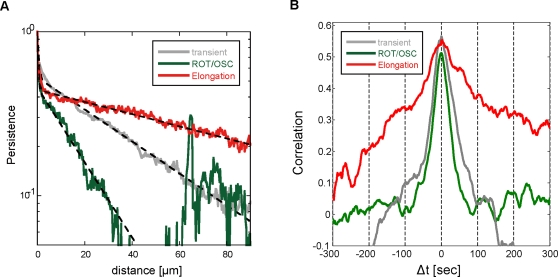
Orientational correlation of ordered patterns and cell movement in polarized cells. (A) Persistence of directional movement as a function of distance in WT STA cells. Red: elongation pattern, green: rotation and oscillation patterns (ROT/OSC), gray: transient pattern among different patterns or random (transient). The fitting curve is the double exponential function (black dash line). (B) Identical angle cross-correlation function for each pattern in WT STA cells. Red: elongation pattern, green: ROT/OSC, gray: transient.

We further investigated the correlation between the ordered pattern and cell movement by examining the persistence length on each pattern in the WT STA cells. The average persistence length of elongating WT STA cells (25 µm) was larger than that of rotating or oscillating cells (9 µm) (Fig. 7A). The dependency of the persistence on the type of ordered patterns suggests that key proteins related to the pattern regulate the location of F-actin accumulation and then steer cell movement. Moreover, we examined this pattern-oriented migration again by comparing iaCFs between the elongation pattern and rotation/oscillation pattern. The iaCF of the elongation pattern showed a long-lasting positive correlation within −300 sec<Δ*t*<300 sec, while the iaCF of the rotation/oscillation pattern rapidly decayed and eventually reached to 0 near Δ*t* = ±100 sec (Fig. 7B). This result means that the elongating cells maintain the elongated cell shape in the direction of motion, and rotating or oscillating cells rapidly turn in accord with the deformation of cell shape. Thus, the ordered patterns of cell shape are coordinated with cell movement even in the absence of external stimuli. The coordination was observed solely in WT STA cells, and, as a separate observation, the asymmetric localization of PTEN occurred in WT STA cells. This suggests that spontaneous cell polarization assisted with the reciprocal localization of PI3K and PTEN, which in turn was responsible for the strong correlation between the patterns and cell migration.

## Discussion

In this study, we demonstrated that the ordered patterns of cell shape, which are dissected into three patterns, are observed in both vegetative and starved cells. A previous study reported rotation and oscillation patterns in starved cells [2]; however, the elongation pattern in WT STA and the ordered patterns in WT VEG cells have not been reported. Because there have been few studies investigating the morphological dynamics of vegetative cells, whether these three ordered patterns are common to different developmental stages or specific only to starved *Dictyostelium* cells remains to be determined. We here demonstrated that these three patterns are also observed in VEG cells, meaning that the timing and direction of pseudopod extension-retraction is not random but organized into ordered patterns even in VEG cells. Our result encourages us to reexamine the conventional notion of spontaneous membrane dynamics in *Dictyostelium* cells, which is that in the absence of a chemoattractant gradient, unpolarized *Dictyostelium* cells extend pseudopodia in more or less random directions.

Recently, Li et al. examined the persistent cell locomotion of *Dictyostelium* cells using long term observations and statistical analysis of centroid locations [26]. They identified that the amoeboid motion could be decomposed into the several characteristic motions: the circular motion of approximately 36 degree/min (CIRC), the oscillation motion with period of 2 to 3 min (OSC), and the instantaneous large turn. We could not identify the instantaneous large turn in our data, but found the cells moving in a fashion similar to CIRC and OSC: The cells in the rotation pattern of cell shape moved in a fashion with angular speed of 36 to 45 degree/min (n = 13; Fig. S5). Moreover, the duration of the rotation pattern was 10 to 20 min that was comparable to that of CIRC. In addition, the oscillation pattern of cell shape showed the period of 2.5 to 3.5 min that was approximately equivalent to that of OSC (2 to 3 min). According to these comparisons, we suppose that the characteristic motions of CIRC and OSC were attributed to the coordination of cell movement with the rotation and oscillation patterns of cell shape, respectively.

We mention that OSC showed the various durations from minutes to an hour though the oscillation pattern of cell shape was maintained for 10 to 20 min but not an hour. We consider that this is because the number of our measurements was not enough to find the oscillation pattern of an hour.

### Mechanism of spontaneous cell migration: coordination with ordered patterns of cell shape

Although the mechanism by which PTEN and PI3K create the ordered patterns of cell shape is still unclear, the observed patterns have enabled us to propose a model of spontaneous cell migration, i.e., the coordination of migration with the formation of ordered patterns of cell shape. This coordination is strengthened especially in WT STA cells due to spontaneous cell polarization mediated by asymmetric PTEN localization. PTEN is asymmetrically localized in WT STA cells, while PTEN is uniformly distributed at the cell membrane in WT VEG cells. The asymmetric localization of PTEN is not necessary for creating ordered patterns but is required for persistently enforcing the direction of movement along with the orientation of cell shape.

Elongating WT STA cells displayed the long-lasting correlation in iaCF, while rotating or oscillating ones showed the rapid decrease of correlation in iaCF. This result suggests that each local pseudopodia extension mediated by PI3K dependent F-actin polymerization enables a cell not only to move forward but also to turn to the left or right. Spontaneous cell migration in polarized cells thereby results from mutual coordinating behavior between ordered patterns and cell movement. We further asked a question regarding the temporal hierarchy between the ordered pattern and cell movement: Does a cell elongate in the direction of cell movement or move in the direction of protrusions? Or do these two processes coordinate instantly? In order to infer this temporal hierarchy, we examined the point at which iaCF shows the maximum value. Given that a cell elongates in the direction of motion with a time delay of τ after cell movement, iaCF exhibits the maximum of correlation at Δ*t = τ*. We found that iaCF showed the maximum correlation at Δ*t = τ = 0* (Fig. 6E). This observation indicates that the coordination between cell shape and cell movement occurs instantly.

### Relationship between the ordered patterns and chemotactic behavior

Our results show that ordered patterns of cell shape are mediated by PI3K and PTEN. PI3K and PTEN are also essential for cell polarity during chemotaxis. The responsibility of the two proteins raises a question as to what relations exist between the ordered patterns and cell polarity in chemotaxis. During chemotaxis, directional sensing by CRAC occurs through the activations of G-protein coupled receptors, and then the localizations of PI3K and PTEN are induced by extracellular chemotactic stimuli. Guidance signals are subject to amplification and feedback regulation of cell polarity, probably to permit the detection of small differences in a shallow gradient. In the case of spontaneous cell migration, Sasaki et al. have found that PI3K and Ras are autonomously activated in the absence of chemoattractants at the sites where F-actin projections emerge, which suggests that spontaneous cell polarity would result from the stochastic activation of the guidance pathway G-protein-independent guidance pathways [12].

We observed that PTEN was localized spontaneously at the rear edge in WT STA cells, and PI3K-dependent F-actin polymerization occurred at both the leading edge and at the rear edge. The elongation pattern indicated that the cells amplify shallow gradients of spontaneously produced PIP_3_ and maintain cell polarity through PI3K and PTEN. Moreover, the switching of ordered patterns suggests that cells stochastically choose one of the three ordered patterns and change it to another pattern in order to ensure overall random exploration during spontaneous cell migration. In contrast, chemotactic cells dominantly select the elongation pattern due to signals from G-proteins and then move toward the source of the chemoattractant by coordinating cell movement with the selected elongation pattern. Hence, cell polarity in chemotaxis could be considered as the property that cells dominantly choose an elongation pattern.

Recent studies have shown that mutants lacking PI3K and/or PTEN do not sustain a polarized cell shape but can exhibit precise chemotaxis [14], [15]. Although cell polarity that is sustained by PI3K and PTEN in chemotaxis could be related to the elongation pattern of cell shape, neither the cell polarity nor the elongation pattern may be necessary for chemotaxis.

### Biological function of the transition of ordered patterns of cell shape

We observed that the ordered patterns of cell shape stochastically transit from one pattern to another. This stochastic transition could add randomness to the motion of *Dictyostelium*. In terms of the advantage of randomness, we note similarities between the stochastic change of flagella rotations of bacteria and the stochastic transition of ordered patterns of *Dictyostelium* cells. Motile bacteria such as *Escherichia coli* (*E.coli*) have flagella that are rotating structures driven by the proton motive force. Although *Dictyostelium* and *E coli* do not use identical locomotory apparatus, both forage with the aid of stochastically switching between two characteristic states, a fairly straight motion and a turning motion. For the fairly straight motion, bacteria rotate flagella in a clock-wise manner, and *Dictyostelium* cells maintain the elongation pattern of cell shape for at least 10 min. Moreover, for the turning motion, bacteria tumble by counter-clockwise rotation of flagella, and *Dictyostelium* cells change into the rotation or oscillation pattern that lasts the order of 10–20 min. Stochastic switching of these ordered patterns allows cells to do overall random exploration.

Regarding the efficiency of searching behavior, Li et al. have conducted the numerical simulation of the amoeboid motion in which a cell finely directs the motion with a persistent time of approximately 9 min [26]. By comparing to the random walk (defined as the motion whose direction and speed are randomly chosen), they found that the fairly straight motion of 9 min in the amoeboid motion allows cells to cover more area in a given time. This non-random motion results in the 1.6 to 2.4 fold increase of the efficiency greater than random walk. In our study, the coordination of the ordered patterns with cell movement suggests that the ordered patterns of cell shape attribute to the non-random motion in foraging behavior, e.g., the straight motion directed by the elongation pattern could realize the fairly straight motion. Based on the discussion done by Li et al, such a non-random (persistent) motion directed by the ordered pattern could improve the efficiency of searching behavior.

In this study, we found the ordered patterns of cell shape were mediated by PI3K/PTEN/F-actin, and the coordination of these patterns with cell movement constituted the mechanism of spontaneous cell migration. Wave-like protrusions have been observed in various cells from flies to mammals [13], [27]–[29]. We believe that the ordered patterns mediated by PI3K/PTEN/F-actin may occur in other cells and that the application of the present approach offers the opportunity to complement the analysis of processes such as leukocyte migration [30]–[32].

## Materials and Methods

### Strains and Cultures

Wild-type, *pten* ^−^, PTEN-GFP, CRAC-GFP, ABD-GFP of AX-2, and *pi3k*1/2^−^ of AX-3 *Dictyostelium discoideum* were cultivated in liquid nutrient medium (HL-5 medium) with appropriate antibiotics and harvested axenically during exponential growth. Cells are at vegetative (VEG) state, which is termed as VEG cells, in the rich HL-5 medium. After 6 to 7 hour of nutrient depletion, VEG cells differentiate into the different developmental stages, starved state. We name the cells at the STA state as starved (STA) cells. We prepared VEG and STA cells used for our measurements as follows: VEG cells were suspended in 10 ml phosphate buffer (PB) at a density of 10^3^ cells/ml, and 1.5 ml of the suspension was placed onto a 1.5% agar plate (66 mm diameter, Falcon). The agar plate was incubated for 30 min to allow the cells to adhere to the agar surface (Nakarai). STA cells were prepared the following conventional protocol. VEG cells were washed 3 times with PB and then plated without nutrient at a density of 5×10^3^ cells/cm^2^ for 6–7 h at 22 C°, and then washed twice by PB. All the experiments were performed at room temperature (22 C°).

We added the PI3K inhibitor LY294002 (Sigma) to a final concentration of 100 µM 30 min before the real-time recording. No differences were observed if the drug was added either 30 min or 60 min before observation. Cells treated with LY294002 and the *pi3k*1/2^−^ mutants tend to detach from the substrate during the extension of pseudopodia. In order to prevent detachment, we placed a 1.0% agarose sheet (TaKaRa) onto PI3K-inhibited cells.

### Digital image processing

The experimental microscope setup consisted of a Leica microscope equipped with a cooled CCD camera (ORCA-AG, Hamamatsu Photonics) with a×20 objective. Time lapse-images were captured with a time interval of 1 sec. We used the phase contrast equipment in conjunction with Metamorph software (Universal Imaging Corporation). The shutter speed of CCD camera was 31 msec. We first flattened the background noise using Metamorph, and then a two-component Gaussian mixture model was fitted to the pixel intensity distribution of each image using the Expectation Maximation algorithm. From this model, a time-dependent intensity threshold was calculated.

### Circular mapping

From each binarized image, we define the position of the centroid as 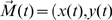. In order to calculate the dynamics of the cell shape in a 2D Cartesian system, we use circular mapping: the radial amplitude of extensions from the centroid to the cell edge are calculated for each image. We define this length value as *Amp*(*θ,t*). Further we define velocity as the displacement of the centroid, 
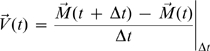
 at time interval 15 s. We also require 2 additional rules to avoid calculation difficulties. First, if the function of cell contour becomes a multivalued function, we employed the value furthest from the centroid. Second, if we obtain an image in which the centroid does not locate within a cell, we do not calculate any values but just adopt the previous values. Since we obtained the ordered patterns when we adopted arc length instead of angle *θ*, these rules did not affect our conclusions.

Actin-binding domain fused GFP (ABD-GFP) was expressed for the observation of F-actin assembly and localization in AX-2 WT cells [25]. We performed fluorescence and phase-contrast measurements every 6 s. There was a 2 s delay between fluorescent measurements and phase-contrast image capture. At each time point, the cell position was identified on the phase-contrast images, and calculated as *Amp*(*θ,t*). We then analyzed the fluorescence intensity data *I*(*r,θ,t*) where *r* represents the length from the centroid. Because F-actin is accumulated nearby cell periphery not but just on the edge, measuring *I*(*r,θ,t*) along cell membrane did not work well. Instead, we employed the variable *Act*(*θ,t*), which is defined as 
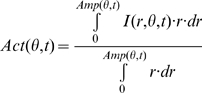
 . The variable *Act*(*θ,t*) is the weighted F-actin concentration by the distance from centroid *r* along the radius in the direction *θ* and at time *t*, normalized by the sum of *r*. *Act*(*θ,t*) is the reliable measure of F-actin accumulation (Fig. S6).

### Correlation analysis

We define the auto-correlation functions of *Amp*(*θ,t*) and *Act*(*θ,t*), and the cross-correlation function between *Act*(*θ,t*) and *Amp*(*θ,t*) as
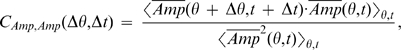


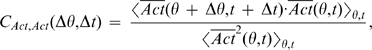


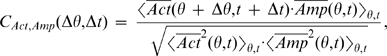
where 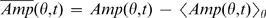 and 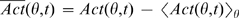. Averages over time and over angles are denoted 〈 〉*_θ,t_*. The range of calculation is the entire period of measurement in time and from 0° to 360° in space.

Further we investigated the relation between velocity 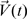 and morphological dynamics *Amp*(*θ,t*) with the identical angle cross-correlation function (iaCF) that is defined as
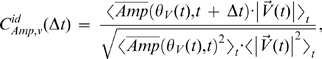
where 
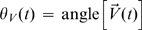
. We obtained the same results on iaCF without the gaussian filtering of velocity field.

In order to evaluate the coordination between cell shape and movement, we calculated the probability of the deviation of *Amp*(*θ_V_*(*t*),*t*) from the mean 〈*Amp*(*θ,t*)〉*_θ_* described as 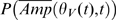, where *θ_V_*(*t*) is the direction of cell movement at an interval of 15 s.

We performed statistical analysis using two-tailed Student's t- test or an analysis of variance (ANOVA). All data found to be significant by ANOVA were compared using Tukey-Kramer test.

### Spectrum analysis of cell shape

In addition to cross-correlation function between *Amp*(*θ,t*) and *Act*(*θ,t*), we further examined the specialized role of PI3K and PTEN in the control of cell shape by using power spectrum of *Amp*(*θ,t*). We calculated the power spectrum of *Amp*(*θ,t*) by using
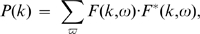
where *F*(*k,ω*) is the 2-dimensional fourier transform of *Amp*(*θ,t*), and F^*^(*k,ω*) is the conjugate of *F*(*k,ω*). *k* describes the spatial frequency of morphological dynamics and *ω* describes temporal frequency. This power spectrum allows us to reveal the long-range correlation of dynamic remodeling of cell shape.

### Clustering analysis

We used clustering method in order to dissect the observed patterns reliably. We can qualitatively dissect elongation, rotation, and oscillation patterns into the combination of the left-hand and the right-hand waves rotating around a circle. For rotating waves, we describe:
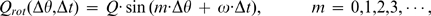
where *m* is the mode number, *ω* is the rate of rotation, *Q* is the centrifugal amplitude. Oscillation is described by superimposition of left-handed and right-handed rotation functions of *Q_rot_*(Δ*θ*,Δ*t*) 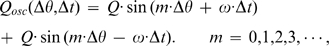
Both functions *Q_rot_*(Δ*θ*,Δ*t*) and *Q_osc_*(Δ*θ*,Δ*t*) may be combined to give:
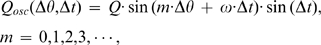
which describes oscillation as waves rotating around a circle. Here, we approximate an obtained ACF, *C_Amp,Amp_*(Δ*θ*,Δ*t*), by the following function: 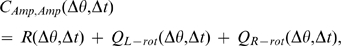


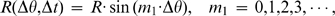


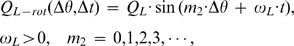


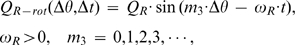
where *m_1_*, *m_2_*, *m_3_* are the mode numbers, *ω_1_* and *ω_R_* are the rates of rotation respectively, and *R*, *Q_L_*, *Q_R_* represent the centrifugal amplitude. *R*(Δ*θ*,Δ*t*) is the coherent component of the vector length from centroid to a point on the basal circle, which is describing elongation pattern. The superposition of three functions results in a qualitative description of the patterns in ACF.

We can obtain both the rates of rotation and the wave amplitudes of *R*(Δ*θ*,Δ*t*), *Q_L-rot_*(Δ*θ*,Δ*t*), and *Q_R-rot_*(Δ*θ*,Δ*t*) by two-dimensional Fourier transform of *C_Amp,Amp_*(Δ*θ*,Δ*t*). The amplitude of each Fourier coefficient represents how large a given spatiotemporal wave contributes to the overall dynamics of cell shape. We note that the amplitudes of the spatial harmonics would differ depending on the size of coordinates. In fact, the patterns of ACF are the superimpositions of various waves with the inherent rate of rotation. For simplicity, we focus on and choose the mode with the largest wave amplitude among standing waves, the one among left-handed waves, and the one among right-handed waves, respectively. Hence, we obtain the parameters of the wave amplitudes *A_S_* = max(*R*), *A_L_* = max(*Q_L_*) and *A_R_* = max(*Q_R_*). Hierarchical clustering was performed on the subsets of 53 and 53 cells in WT VEG and WT STA cells, respectively. We use three parameters *A_S_*, *A_L_*, and *A_R_* for the clustering. However, the flip horizontal of *C_Amp,Amp_*(Δ*θ*,Δ*t*) sometimes affects the result of clustering. To avoid this artificial problem, we define *A_M_* and *A_M_* by sorting *A_L_* and *A_R_* as *A_M_* = max(*A_L_*,*A_R_*) and *A_M_* = min(*A_L_*,*A_R_*). This treatment allows us to obtain the robust clusters (Fig. S7). Clustering was done by means of a standard single-linkage algorithm with a Euclidean metric (Matlab, Mathworks).

### Persistence of directional movement

We defined persistency of moving direction as 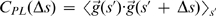, where *s* represents the distance of the trajectory from the starting point (*t* = 0), and 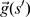 represents the tangent vector at *s* = *s′.* We also defined persistence length *l_C_* as *C_PL_*(*l_C_*) = *e^−1^*. Persistence length is a preferable measure for determining for how far a cell typically moves straight in a given direction.

## Supporting Information

Text S1 Supplementary results and discussions
(0.10 MB DOC)

Figure S1 Long-term measurement of the morphological dynamics of cell shape.We measured a single WT vegetative cell for 3.3 h and then calculated the autocorrelation function of *Amp*(*θ, t*) at each time window (500 s). Six examples of autocorrelation function are shown on the left side of *Amp*(*θ, t*). We found that the ordered pattern dynamically changes; for instance, from rotation to oscillation.
(0.40 MB PDF)

Figure S2 Multiple pseudopodia due to loss of PTEN. Typical *pten* ^&minus^ cells in VEG state. White arrowheads represent irregular pseudopodia. Scale bar is 10 µm. The number indicates time of measurement [second].
(0.04 MB PDF)

Figure S3 PI3K inactivation reduces the amplitude of pseudopodia. Average power spectra of cell morphology. Upper: WT cells (red solid line), WT+LY294002 cells (green dash line) and *pi3k*1/2^&minus^ cells (green solid line). Lower: *pten* ^&minus^ cells (red) and *pten* ^&minus^+LY294002 cells (green). Left is VEG state and right is STA state. The individual power spectra of either WT cells or *pten* ^&minus^ cells (pale red) and those of PI3K-inhibited cells (pale green) were plotted. All averaged power spectra were well fitted by *P(k)*&prop*k* ^−1.7^ (black dash line).
(0.12 MB PDF)

Figure S4 Characterization of the centre of mass displacements. (A) Average mean square displacement along the trajectory as a function of time and (B) autocorrelation function of instantaneous velocity for WT (red), *pten* ^&minus^ (blue) and *pi3k*1/2^&minus^ (green) in both the VEG and STA states. All curves of the MSD fit a decaying power-law. We adopt time interval of 1 s (for MSD) and 5 s (for the autocorrelation function of velocity) in calculating the center of mass displacements, respectively.
(0.10 MB PDF)

Figure S5 Trajectory of centroid and angular dynamics of cell movement Upper column: trajectory of each ordered pattern. Red asterisk represents the start point. Middle column: the angular dynamics of cell movement of each ordered pattern. Lower column: the corresponding ordered patterns
(0.69 MB PDF)

Figure S6 *Act*(*&theta, t*) : a reliable measure of F-actin accumulation. (A) We employed *Act*(*&theta, t*) instead of the measure of *I*(*r, &theta, t*) along cell membrane because F-actin accumulates nearby cell membrane but not on the edge of cell membrane (see Figure 5A). To test the reliability of *Act*(*&theta, t*), we compared it with the largest intensity of F-actin along a radius from centroid, *Imax*(*&theta, t*). *Act*(*&theta, t*) is proportional to *Imax*(*&theta, t*). (B) A example of *Amp*(*&theta, t*) (oscillation pattern). (C) *Act*(*&theta, t*) (left) and *Imax*(*&theta, t*) (right) of the example presented in (B). (D) Cross-correlation functions (CCF). (left) CCF between *Act*(*&theta, t*) and *Amp*(*&theta, t*), (right) CCF between *Act*(*&theta, t*) and *Imax*(*&theta, t*). The similarity of CCFs indicate that *Act*(*&theta, t*) is a reliable measure of F-actin accumulation as well as *Imax*(*&theta, t*).
(0.41 MB PDF)

Figure S7 Clustering analysis of autocorrelation function. We first subject autocorrelation function (ACF) to Fourier transform to obtain the three parameters, *AS*, *AL*, and *AR*. We then conduct clustering analysis of ACF based on the obtained parameters. We show the clustering tree of wild-type vegetative cells.
(0.37 MB PDF)

Movie S1 Elongating WT vegetative cell.
(0.27 MB MOV)

Movie S2 Rotating WT vegetative cell.
(0.36 MB MOV)

Movie S3 Oscillating WT vegetative cell.
(0.24 MB MOV)

Movie S4 Elongating WT starved cell.
(0.23 MB MOV)

Movie S5 Rotating WT starved cell.
(0.24 MB MOV)

Movie S6 Oscillating WT starved cell.
(0.37 MB MOV)

Movie S7 pten-vegetative cell exhibiting a random membrane dynamics.
(0.36 MB MOV)

Movie S8 pten-starved cell exhibiting a random membrane dynamics.
(0.28 MB MOV)

Movie S9 LY294002-treated WT vegetative cell exhibiting a random membrane dynamics.
(0.69 MB MOV)

Movie S10 LY294002-treated WT starved cell exhibiting a random membrane dynamics.
(0.66 MB MOV)

Movie S11 pi3k1/2-vegetative cell exhibiting a random membrane dynamics.
(0.43 MB MOV)

Movie S12 pi3k1/2-starved cell exhibiting a random membrane dynamics.
(0.71 MB MOV)

Movie S13 LY294002-treated pten-vegetative cell exhibiting a random membrane dynamics.
(0.28 MB MOV)

Movie S14 LY294002-treated pten-starved cell exhibiting a random membrane dynamics.
(0.27 MB MOV)
