# Supplementary material for: Ordered Patterns of Cell Shape and Orientational Correlation during Spontaneous Cell Migration
Source: PLoS One. 2008 Nov 17;3(11):e3734. doi: 10.1371/journal.pone.0003734 (PMC2581918; doi:10.1371/journal.pone.0003734)
Supplement: Text S1 — Supplementary results and discussions (0.10 MB DOC) [file pone.0003734.s001.doc]

# Ordered patterns of cell shape and orientational correlation during spontaneous cell migration

Supplementary information

Yusuke T. Maeda*, Junya Inose, Miki Y. Matsuo,

Suguru Iwaya and Masaki Sano

Department of Physics, the University of Tokyo

7-3-1 Hongo, Bunkyo-ku, Tokyo 113-0033, Japan

* ymaeda@rockefeller.edu

Supporting results and discussions

Examination of the effect of cell-cell interaction via cAMP signaling on the ordered patterns

A fraction of the WT STA cells secrete cAMP into the extracellular environment (S1). The surrounding cells expressing cAMP receptor sense the direction of cAMP, recruit CRAC protein at the leading edge and then move towards the cAMP source. This type of cell-cell interaction may affect the behavior of cell movement. In our experiment, it is highly unlikely that a cell senses a cAMP signal from other cells because we observed individual cells at the very low cell density. In addition, we hardly observed the localization of CRAC-GFP at the leading edge, suggesting that cells did not sense extracellular chemical signals during spontaneous cell migration. Further, we added caffeine, which blocks adenylyl cyclase in order to examine the effect of newly synthesized cAMP on ordered patterns. We observed three ordered patterns in the presence of caffeine and thereby excluded the possible involvement of a directional sensing module in the organization of the ordered patterns. Thus, we conclude that directional sensing was not involved in our experiment.

Characterization of center of mass displacements

We characterized cell movement for a two-dimensional random walker by $\left\langle\Delta r^{2}(t)\right\rangle$, the mean square displacement (MSD), and $C_{V}(t)$, the velocity autocorrelation function (S2,S3). We calculate the MSD as a function of the time interval *t*:

$\left\langle\Delta r^{2}(t)\right\rangle=\left\langle(x(t_{0}+t)-x(t_{0}))^{2}+(y(t_{0}+t)-y(t_{0}))^{2}\right\rangle_{t_{0}}$, (1)

where we take the average over all $t_{0}$. We use the instantaneous velocity to calculate the following velocity autocorrelation function:

$C_{V}(t)=\frac{\left\langle\overrightarrow{V}(t_{0}+t)\cdot\overrightarrow{V}(t_{0})\right\rangle_{t_{0}}}{\left\langle\overrightarrow{V}^{2}(t_{0})\right\rangle_{t_{0}}}$. (2)

Typically, in a diffusive process, MSD is a linear function of time. Anomalous diffusion is a diffusion process with a non-linear dependence on time. We plot $\left\langle\Delta r^{2}(t)\right\rangle$ as a function of time interval (Fig. S4). We obtained in WT STA cells, and in the WT VEG cells, in PI3K-inhibited cells, in *pten* null mutants. These results indicate These results indicate that (i) all types of cell movement are more or less super-diffusive, and that　(ii) cell movement of WT STA cells is strongly super-diffusive rather than those of WT cells and mutants. Super-diffusion is the result of active cellular migrating processes. Moreover, the average MSD of WT STA cells is largest among those of all cell types, suggesting that WT STA cells spread more widely after long time periods.

Next, we plotted $C_{V}(t)$ as a function of time (Fig. S4). The velocity of cell movement was calculated from the center of mass displacement at a time interval of 5 s. The velocity autocorrelation function is useful for investigating persistent behavior of cell movement. After long periods of time, velocities are uncorrelated and the velocity autocorrelation function decreases to zero. We found that a decaying double-exponential curve fits the data well. The $C_{V}(t)$ of WT STA cells decays slowly over time [velocities are uncorrelated at long times $C_{V}(t)$=0], while those of the other cell types rapidly decrease to zero. This result indicates that a WT STA cell moves with a more highly correlated velocity than the others, which is consistent with the result for persistence length (Table 1).

**Legends of supplementary figures**

Figure S1. Long-term measurement of the morphological dynamics of cell shape.

We measured a single WT vegetative cell for 3.3 h and then calculated the autocorrelation function of $Amp(\theta,t)$ at each time window (500 s). Six examples of autocorrelation function are shown on the left side of $Amp(\theta,t)$. We found that the ordered pattern dynamically changes; for instance, from rotation to oscillation.

**Figure S2. Multiple pseudopodia due to loss of PTEN**

Typical *pten*^－^ cells in VEG state. White arrowheads represent irregular pseudopodia. Scale bar is 10μm. The number indicates time of measurement [second].

**Figure S3. PI3K inactivation reduces the amplitude of pseudopodia**

Average power spectra of cell morphology. Upper: WT cells (red solid line), WT+LY294002 cells (green dash line) and *pi3k1/2*^－^ cells (green solid line). Lower: *pten*^－^ cells (red) and *pten*^－^+LY294002 cells (green). Left is VEG state and right is STA state. The individual power spectra of either WT cells or *pten*^－^ cells (pale red) and those of PI3K-inhibited cells (pale green) were plotted. All averaged power spectra were well fitted by $P(k)\propto k^{-1.7}$ (black dash line).

**Figure S4. Characterization of the centre of mass displacements**

(a) Average mean square displacement along the trajectory as a function of time and (b) autocorrelation function of instantaneous velocity for WT (red), *pten*^－^ (blue) and *pi3k1/2*^－^ (green) in both the VEG and STA states. All curves of the MSD fit a decaying power-law. We adopt time interval of 1s (for MSD) and 5 s (for the autocorrelation function of velocity) in calculating the center of mass displacements, respectively.

**Figure S5. Trajectory of centroid and angular dynamics of cell movement**

Upper column: trajectory of each ordered pattern. Red asterisk represents the start point. Middle column: the angular dynamics of cell movement of each ordered pattern. Lower column: the corresponding ordered patterns

**Figure S6. $Act(\theta,t)$: a reliable measure of F-actin accumulation.**

(A) We employed $Act(\theta,t)$ instead of the measure of $I(r,\theta,t)$ along cell membrane because F-actin accumulates nearby cell membrane but not on the edge of cell membrane (see Figure 5A). To test the reliability of $Act(\theta,t)$, we compared it with the largest intensity of F-actin along a radius from centroid, $I_{\max}(\theta,t)$. $Act(\theta,t)$ is proportional to $I_{\max}(\theta,t)$.

(B) A example of $Amp(\theta,t)$ (oscillation pattern).

(C) $Act(\theta,t)$ (left) and $I_{\max}(\theta,t)$ (right) of the example presented in (B).

(D) Cross-correlation functions (CCF). (left) CCF between $Act(\theta,t)$ and $Amp(\theta,t)$, (right) CCF between $I_{\max}(\theta,t)$ and $Amp(\theta,t)$.

The similarity of CCFs indicate that $Act(\theta,t)$ is a reliable measure of F-actin accumulation as well as $I_{\max}(\theta,t)$.

**Figure S7. Clustering analysis of autocorrelation function**

We first subject autocorrelation function (ACF) to Fourier transform to obtain the three parameters, $A_{S}$, $A_{L}$, and $A_{R}$. We then conduct clustering analysis of ACF based on the obtained parameters. We show the clustering tree of wild-type vegetative cells.

**Supporting movies**

Movie S1: Elongating WT vegetative cell.

Movie S2: Rotating WT vegetative cell.

Movie S3: Oscillating WT vegetative cell.

Movie S4: Elongating WT starved cell.

Movie S5: Rotating WT starved cell.

Movie S6: Oscillating WT starved cell.

Movie S7: *pten*^－^ vegetative cell exhibiting a random membrane dynamics.

Movie S8: *pten*^－^ starved cell exhibiting a random membrane dynamics.

Movie S9: LY294002-treated WT vegetative cell exhibiting a random membrane dynamics.

Movie S10: LY294002-treated WT starved cell exhibiting a random membrane dynamics.

Movie S11: *pi3k1/2*^－^ vegetative cell exhibiting a random membrane dynamics.

Movie S12: *pi3k1/2*^－^ starved cell exhibiting a random membrane dynamics.

Movie S13: LY294002-treated *pten*^－^ vegetative cell exhibiting a random membrane dynamics.

Movie S14: LY294002-treated *pten*^－^ starved cell exhibiting a random membrane dynamics.

References

S1. Saran S, Meima ME, Alvarez-Curto E, Weening KE, Rozen DE & Schaap P (2002) *J.Muscle Res.Cell Motil.* **23**: 793-802.

S2. Rieu J-P, Upadhyaya A, Glazier JA, Ouchi NB & Sawada Y (2000) *Biophys. J.* **79**: 1903-1914 (2000)

S3. Shenderov AD & Sheetz MP (1997) *Biophys. J.* **72**: 2382-2389.
