# Supplementary figures and images for: Ordered Patterns of Cell Shape and Orientational Correlation during Spontaneous Cell Migration
Source: PLoS One. 2008 Nov 17;3(11):e3734. doi: 10.1371/journal.pone.0003734 (PMC2581918; doi:10.1371/journal.pone.0003734)

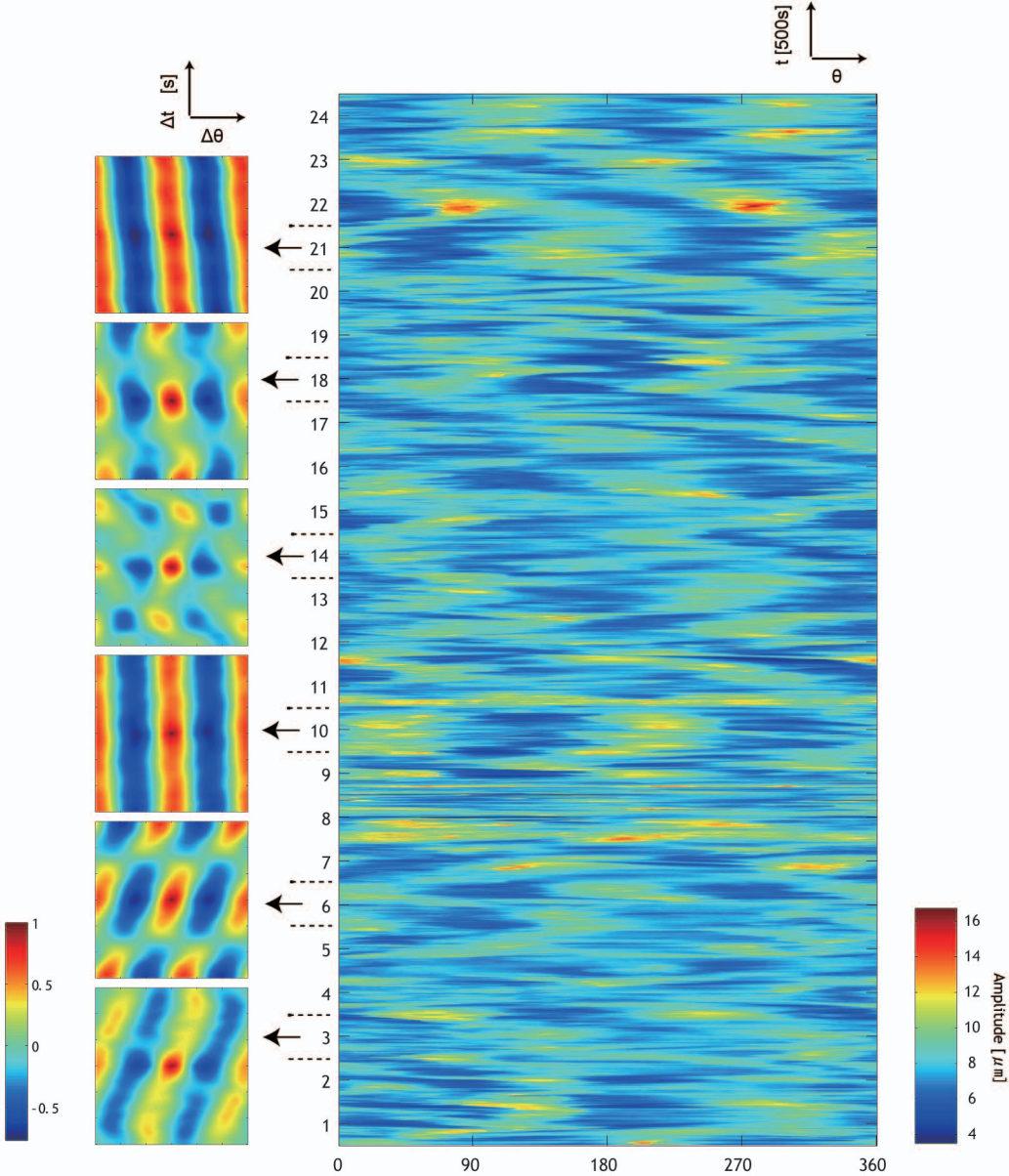

Supplement: Figure S1 — Long-term measurement of the morphological dynamics of cell shape.We measured a single WT vegetative cell for 3.3 h and then calculated the autocorrelation function of Amp(θ, t) at each time window (500 s). Six examples of autocorrelation function are shown on the left side of Amp(θ, t). We found that the ordered pattern dynamically changes; for instance, from rotation to oscillation. (0.40 MB PDF) [file pone.0003734.s002.pdf]

0

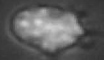

25

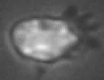

75

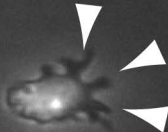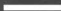

Supplement: Figure S2 — Multiple pseudopodia due to loss of PTEN. Typical pten &minus cells in VEG state. White arrowheads represent irregular pseudopodia. Scale bar is 10 µm. The number indicates time of measurement [second]. (0.04 MB PDF) [file pone.0003734.s003.pdf]

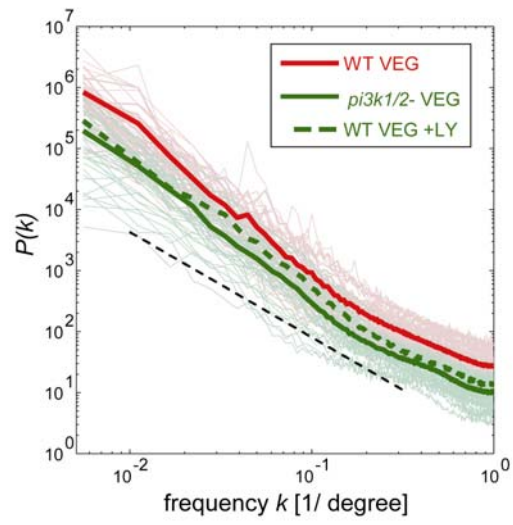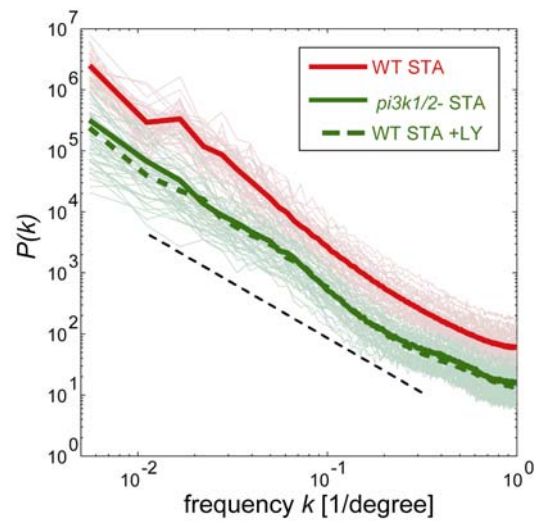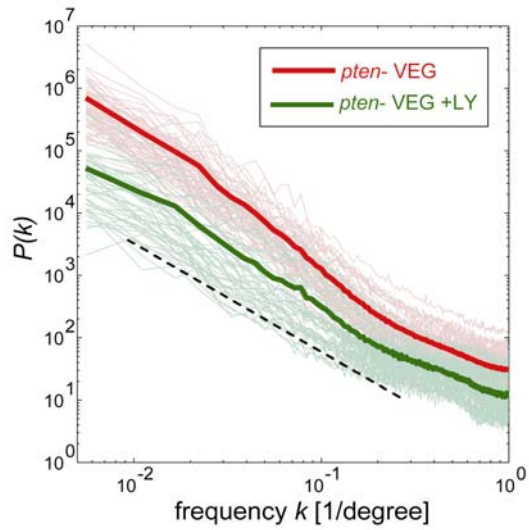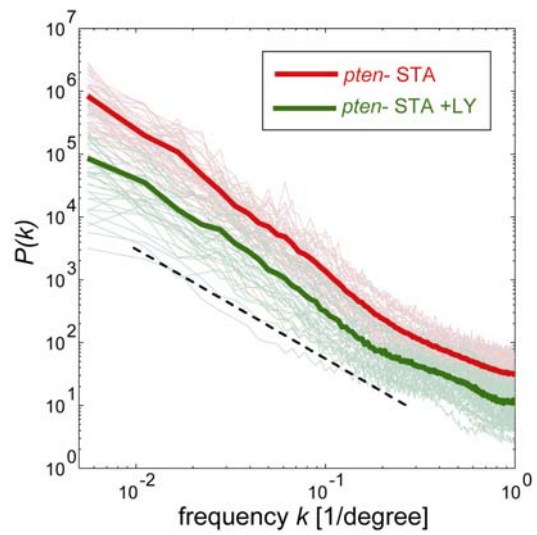

Supplement: Figure S3 — PI3K inactivation reduces the amplitude of pseudopodia. Average power spectra of cell morphology. Upper: WT cells (red solid line), WT+LY294002 cells (green dash line) and pi3k1/2&minus cells (green solid line). Lower: pten &minus cells (red) and pten &minus+LY294002 cells (green). Left is VEG state and right is STA state. The individual power spectra of either WT cells or pten &minus cells (pale red) and those of PI3K-inhibited cells (pale green) were plotted. All averaged power spectra were well fitted by P(k)&propk −1.7 (black dash line). (0.12 MB PDF) [file pone.0003734.s004.pdf]

**a**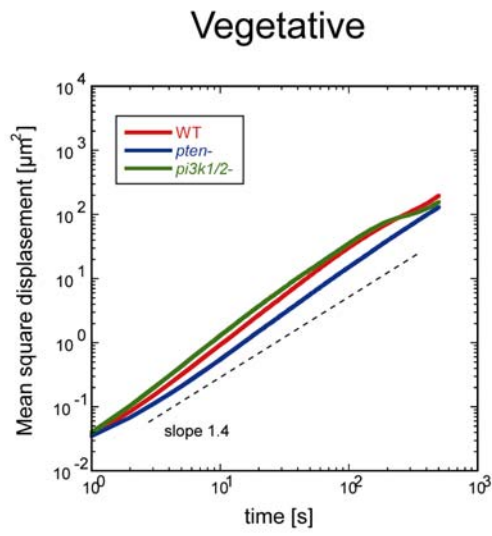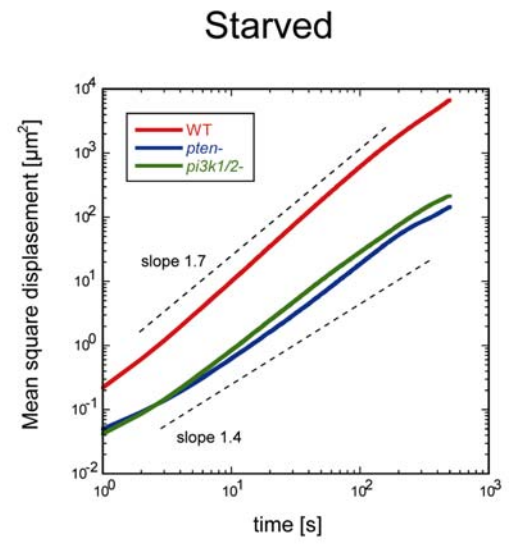**b**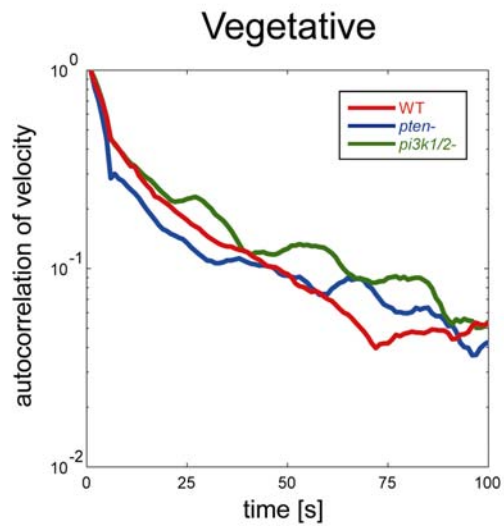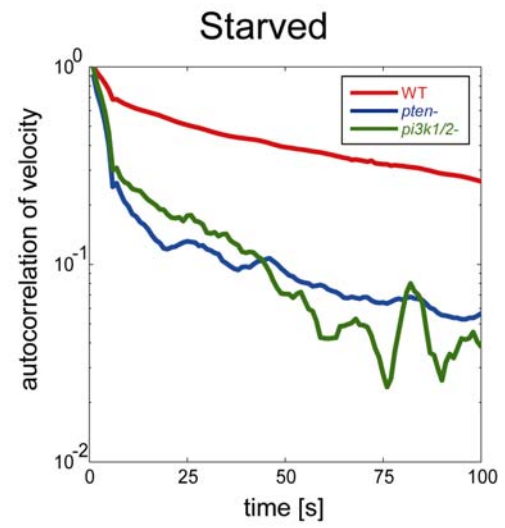

Supplement: Figure S4 — Characterization of the centre of mass displacements. (A) Average mean square displacement along the trajectory as a function of time and (B) autocorrelation function of instantaneous velocity for WT (red), pten &minus (blue) and pi3k1/2&minus (green) in both the VEG and STA states. All curves of the MSD fit a decaying power-law. We adopt time interval of 1 s (for MSD) and 5 s (for the autocorrelation function of velocity) in calculating the center of mass displacements, respectively. (0.10 MB PDF) [file pone.0003734.s005.pdf]

Trajectory

Elongating

Rotating

Oscillating

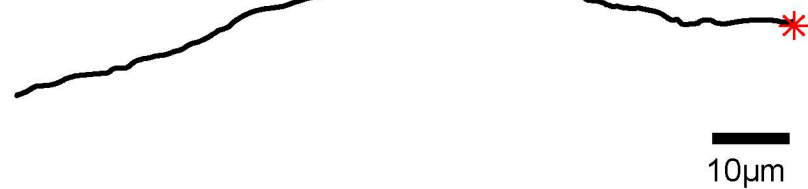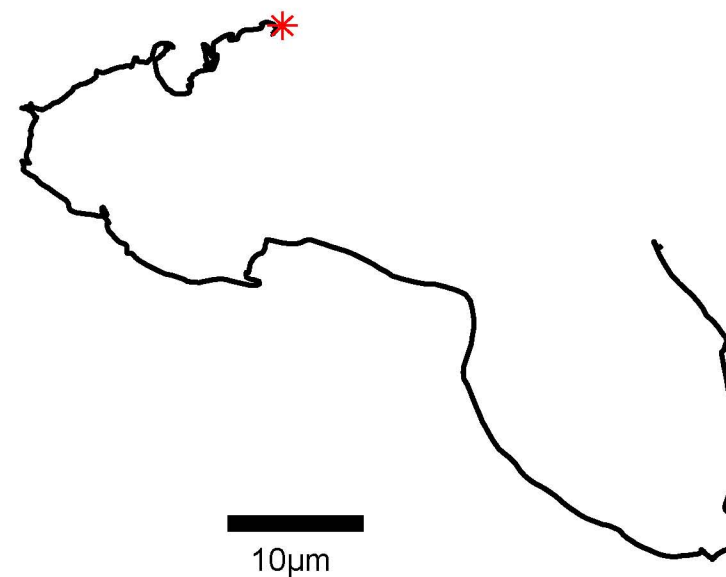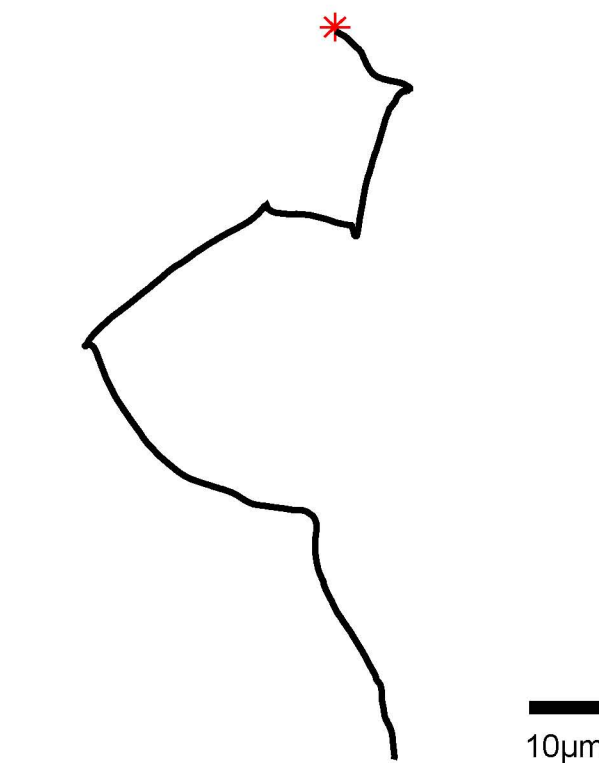

Angle

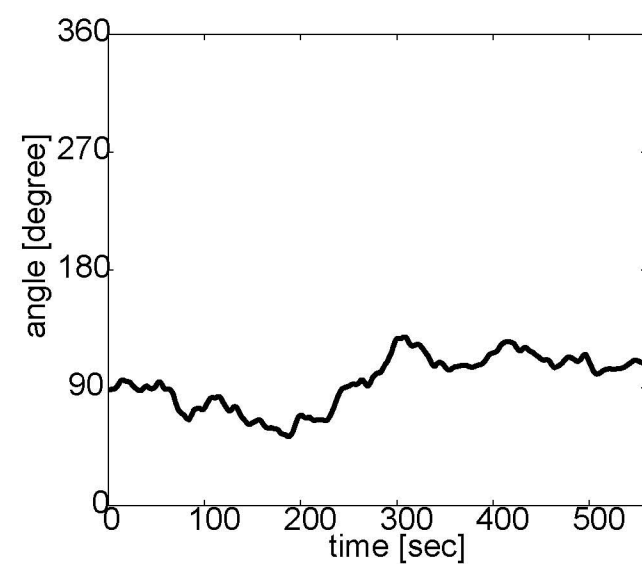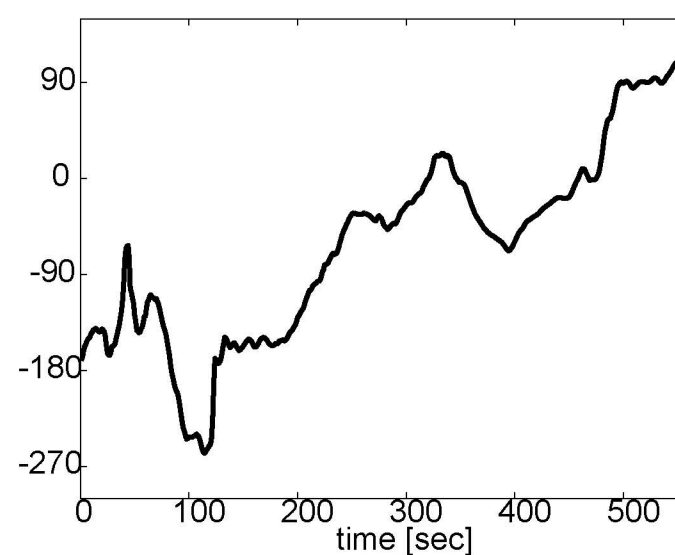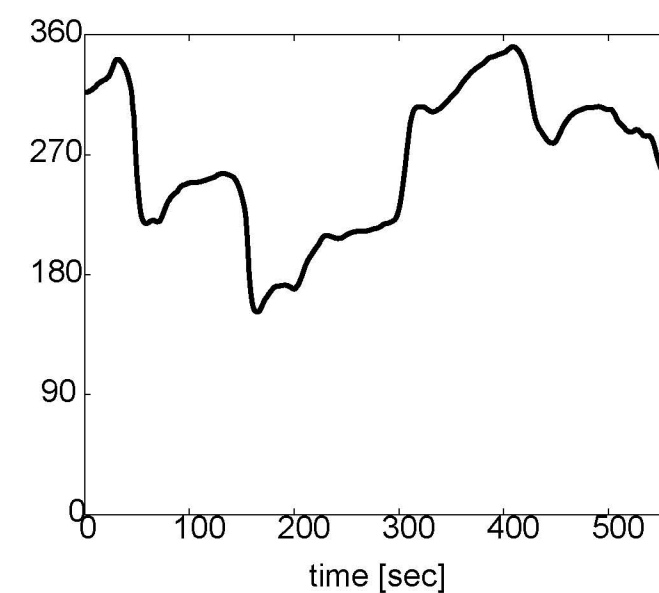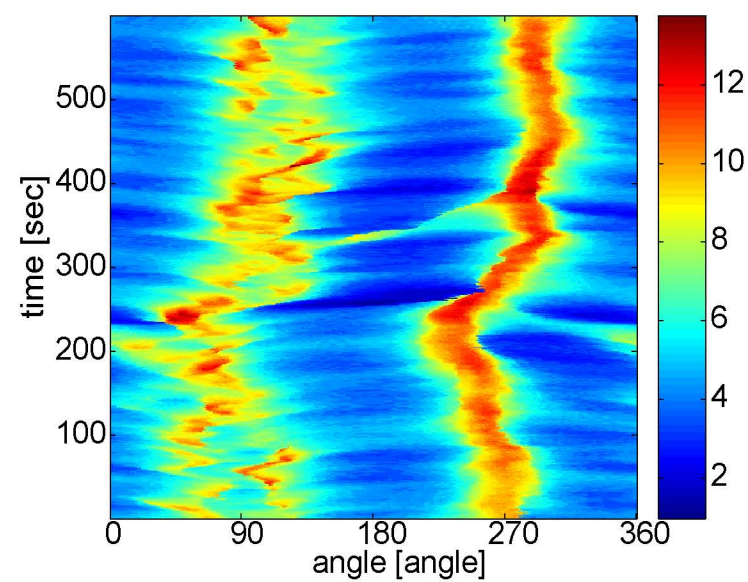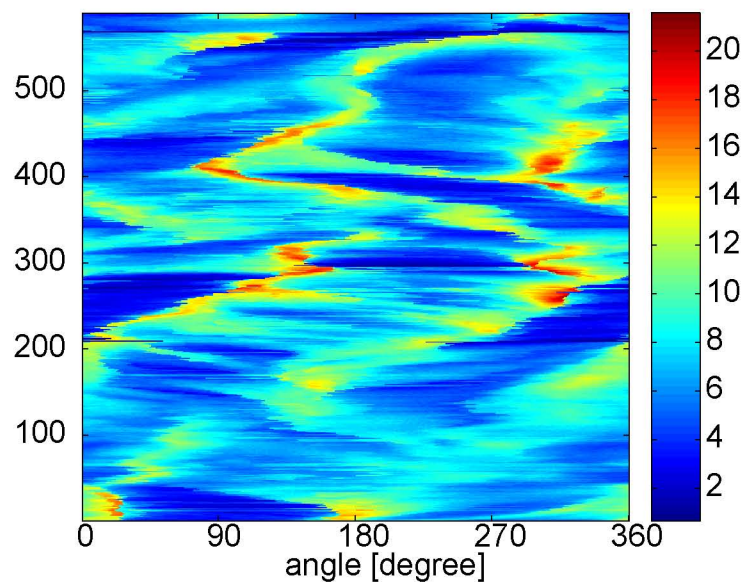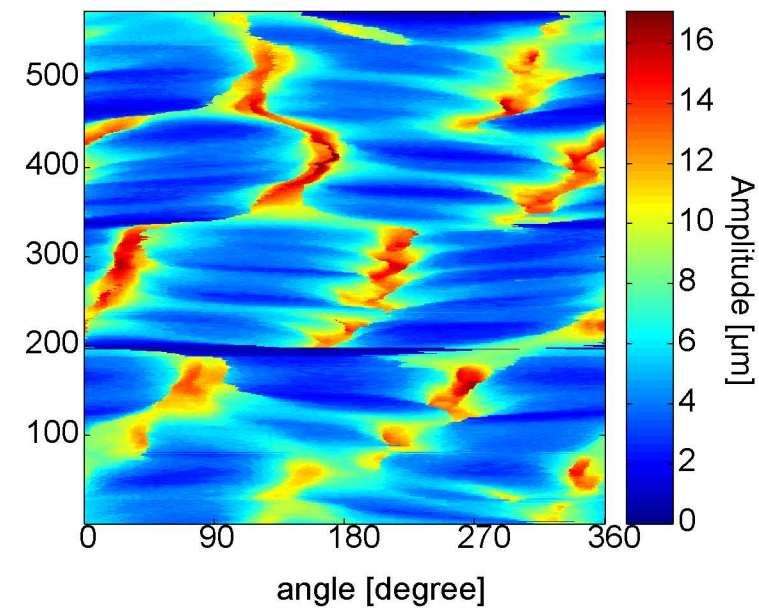

Supplement: Figure S5 — Trajectory of centroid and angular dynamics of cell movement Upper column: trajectory of each ordered pattern. Red asterisk represents the start point. Middle column: the angular dynamics of cell movement of each ordered pattern. Lower column: the corresponding ordered patterns (0.69 MB PDF) [file pone.0003734.s006.pdf]

A

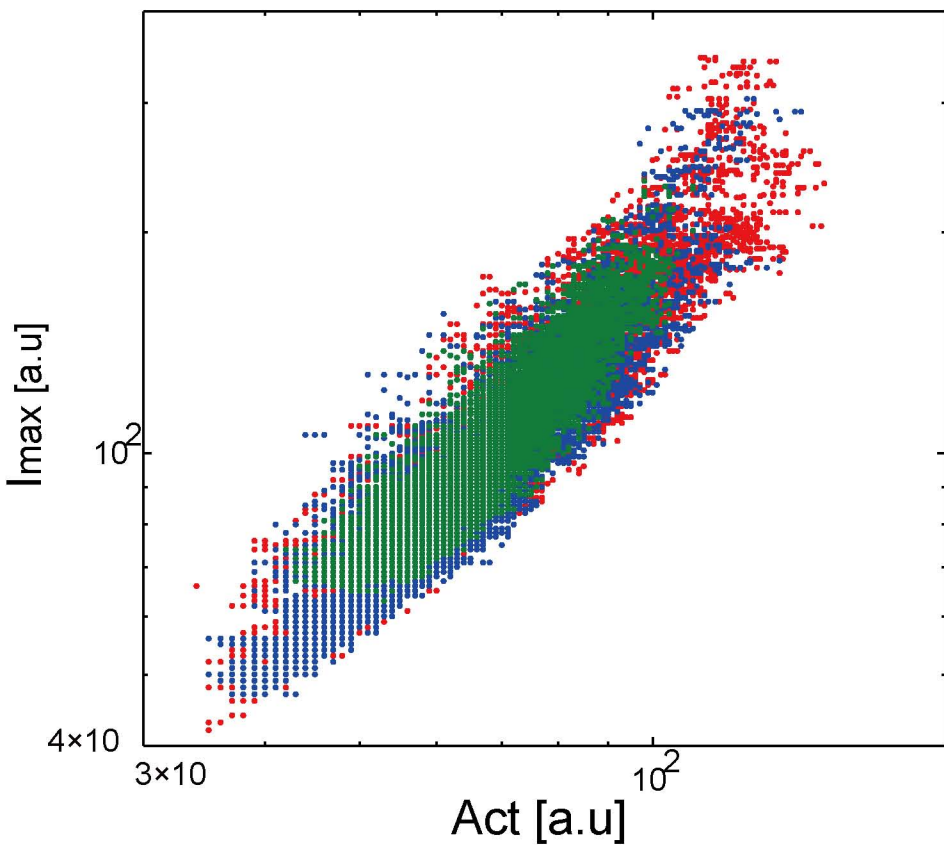

B

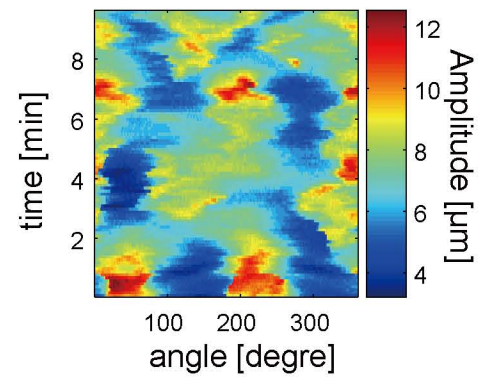

C

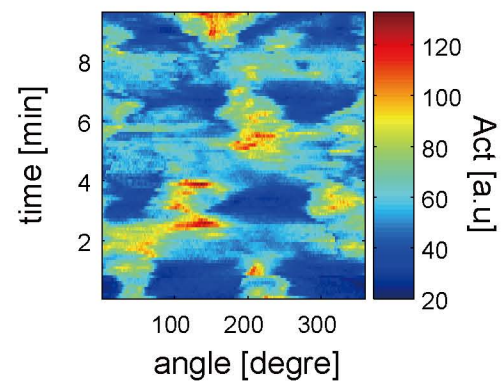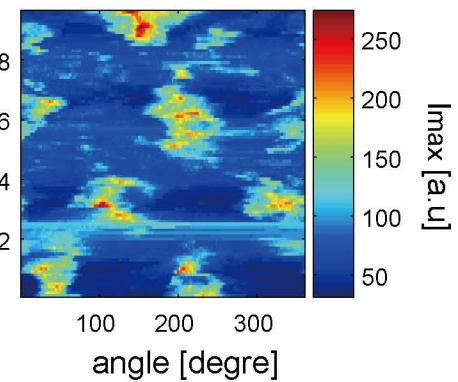

D

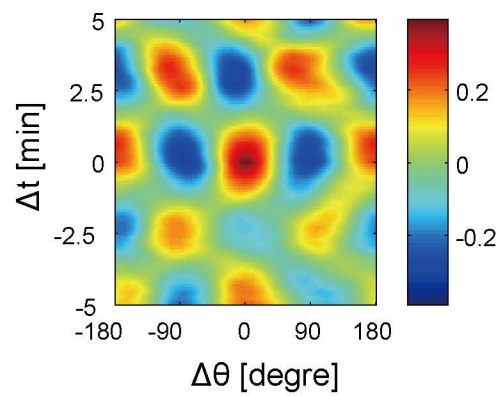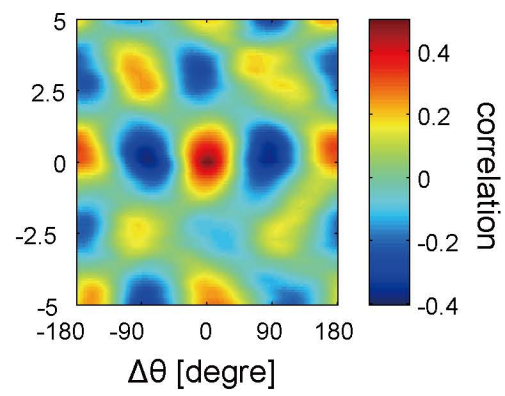

Supplement: Figure S6 — Act(&theta, t) : a reliable measure of F-actin accumulation. (A) We employed Act(&theta, t) instead of the measure of I(r, &theta, t) along cell membrane because F-actin accumulates nearby cell membrane but not on the edge of cell membrane (see Figure 5A). To test the reliability of Act(&theta, t), we compared it with the largest intensity of F-actin along a radius from centroid, Imax(&theta, t). Act(&theta, t) is proportional to Imax(&theta, t). (B) A example of Amp(&theta, t) (oscillation pattern). (C) Act(&theta, t) (left) and Imax(&theta, t) (right) of the example presented in (B). (D) Cross-correlation functions (CCF). (left) CCF between Act(&theta, t) and Amp(&theta, t), (right) CCF between Act(&theta, t) and Imax(&theta, t). The similarity of CCFs indicate that Act(&theta, t) is a reliable measure of F-actin accumulation as well as Imax(&theta, t). (0.41 MB PDF) [file pone.0003734.s007.pdf]

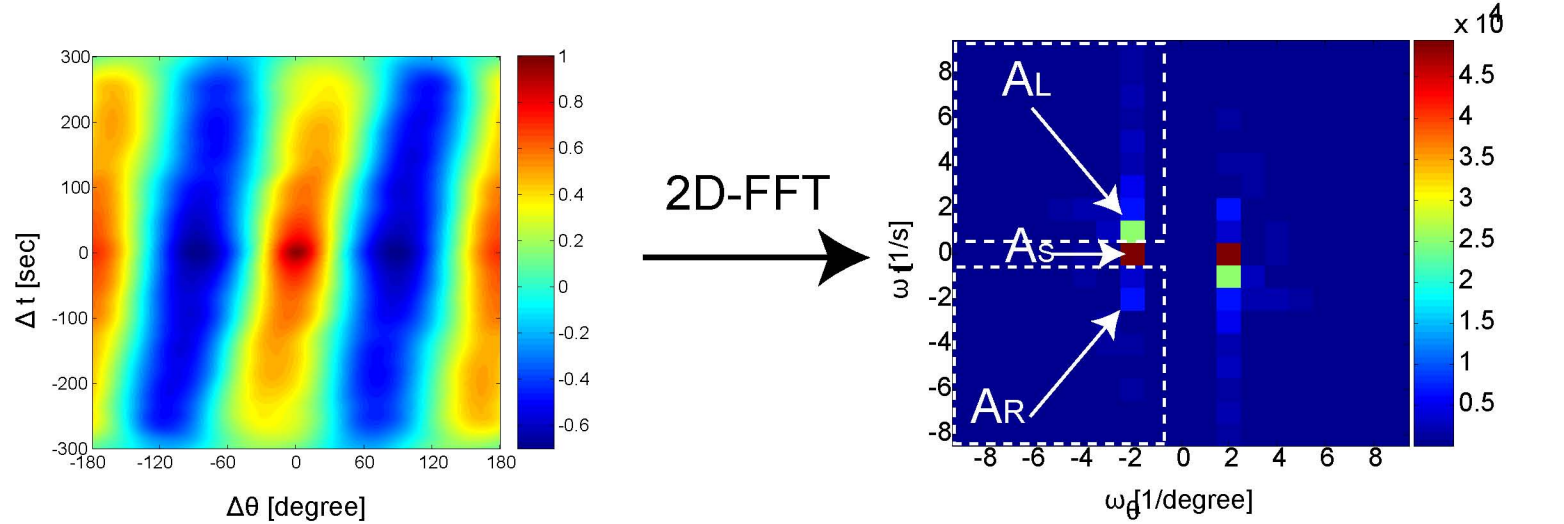

Sorting

$$A_M = \max(A_L, A_R)$$

$$A_m = \min(A_L, A_R)$$

Clustering by  $A_S$ ,  $A_M$ , and  $A_m$

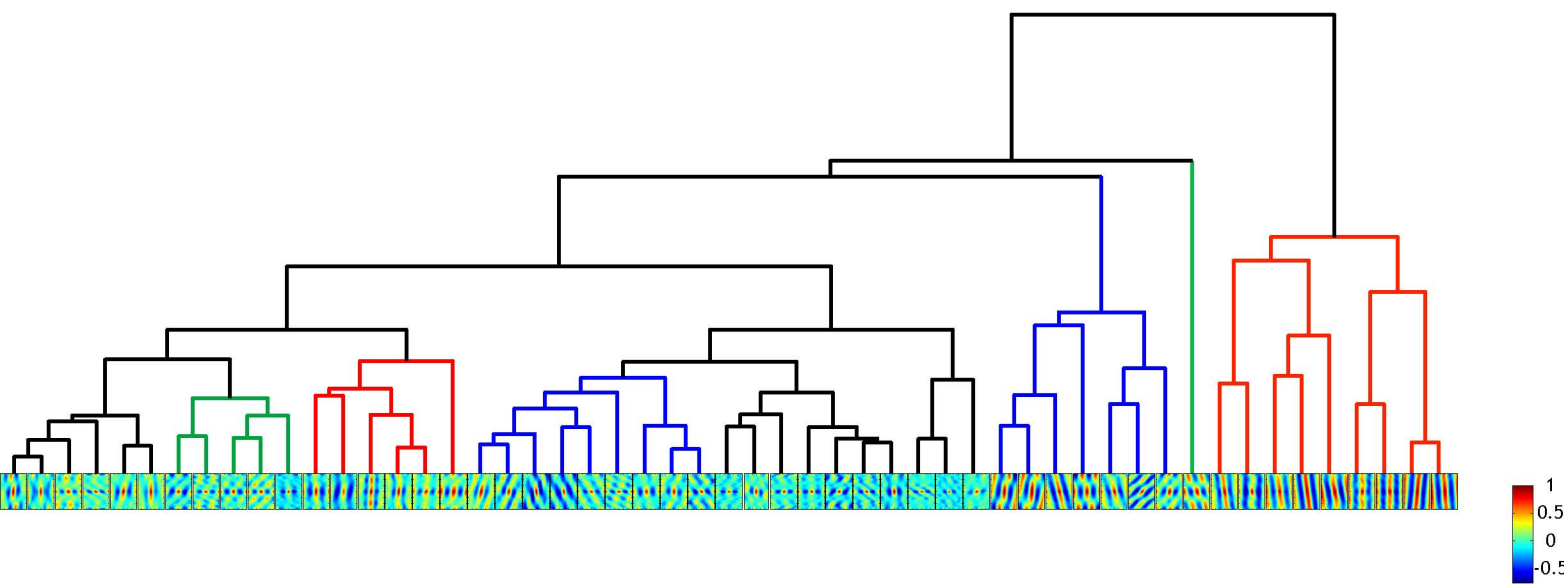

Supplement: Figure S7 — Clustering analysis of autocorrelation function. We first subject autocorrelation function (ACF) to Fourier transform to obtain the three parameters, AS, AL, and AR. We then conduct clustering analysis of ACF based on the obtained parameters. We show the clustering tree of wild-type vegetative cells. (0.37 MB PDF) [file pone.0003734.s008.pdf]
